# Cold Cognition as Predictor of Treatment Response to rTMS; A Retrospective Study on Patients With Unipolar and Bipolar Depression

**DOI:** 10.3389/fnhum.2022.888472

**Published:** 2022-07-25

**Authors:** Reza Rostami, Reza Kazemi, Zahra Nasiri, Somayeh Ataei, Abed L. Hadipour, Nematollah Jaafari

**Affiliations:** ^1^Department of Psychology, University of Tehran, Tehran, Iran; ^2^Department of Cognitive Psychology, Institute for Cognitive Science Studies>, Tehran, Iran; ^3^Convergent Technologies Research Center, University of Tehran, Tehran, Iran; ^4^Department of Neuropsychology, Faculty of Psychology, Institute of Cognitive Neuroscience, Ruhr-University Bochum, Bochum, Germany; ^5^Department of Cognitive Sciences, University of Messina, Messina, Italy; ^6^Unité de Recherche Clinique Intersectorielle en Psychiatrie Pierre Deniker, Centre Hospitalier Henri Laborit, Poitiers, France; ^7^University Poitiers & CHU Poitiers, INSERM U1084, Laboratoire Expérimental et Clinique en Neurosciences, Poitiers, France

**Keywords:** bipolar depression, unipolar depression, transcranial magnetic stimulation (TMS), cognitive predictors, cold cognition, predictors of treatment response

## Abstract

**Background:**

Cognitive impairments are prevalent in patients with unipolar and bipolar depressive disorder (UDD and BDD, respectively). Considering the fact assessing cognitive functions is increasingly feasible for clinicians and researchers, targeting these problems in treatment and using them at baseline as predictors of response to treatment can be very informative.

**Method:**

In a naturalistic, retrospective study, data from 120 patients (Mean age: 33.58) with UDD (*n* = 56) and BDD (*n* = 64) were analyzed. Patients received 20 sessions of bilateral rTMS (10 Hz over LDLPFC and 1 HZ over RDLPFC) and were assessed regarding their depressive symptoms, sustained attention, working memory, and executive functions, using the Beck Depression Inventory (BDI-II) and Neuropsychological Test Automated Battery Cambridge, at baseline and after the end of rTMS treatment course. Generalized estimating equations (GEE) and logistic regression were used as the main statistical methods to test the hypotheses.

**Results:**

Fifty-three percentage of all patients (*n* = 64) responded to treatment. In particular, 53.1% of UDD patients (*n* = 34) and 46.9% of BDD patients (*n* = 30) responded to treatment. Bilateral rTMS improved all cognitive functions (attention, working memory, and executive function) except for visual memory and resulted in more modulations in the working memory of UDD compared to BDD patients. More improvements in working memory were observed in responded patients and visual memory, age, and sex were determined as treatment response predictors. Working memory, visual memory, and age were identified as treatment response predictors in BDD and UDD patients, respectively.

**Conclusion:**

Bilateral rTMS improved cold cognition and depressive symptoms in UDD and BDD patients, possibly by altering cognitive control mechanisms (top-down), and processing negative emotional bias.

## Introduction

Repetitive transcranial magnetic stimulation (rTMS) has been increasingly used as a therapeutic solution for depression for more than a decade and evidence regarding its efficacy has been reported in several meta-analyses (Brunoni et al., 2017; Cao et al., 2018; Mutz et al., 2018). Worth noting is that the United States Food and Drug Administration (FDA) has approved certain rTMS protocols to treat major depressive disorder (MDD) (Berlim et al., 2017; Hung et al., 2020), however, the question remains on how to improve response to this treatment by optimizing stimulation protocols (Cash et al., 2017; Pitkänen et al., 2017; Miron et al., 2021) and developing markers to enable prediction of its therapeutic effects on patients. In that light, predictors of treatment response to rTMS, potentially identifying patients who will likely respond to the intervention, play an essential role in the pursuit to deliver personalized rTMS treatment.

In a recent investigation, response rates of MDD patients have been reported to be somewhere between 58 and 83 percent (Sackeim et al., 2020), while it has been reported to be 41% in a sample of patients with bipolar depressive disorder (Rostami et al., 2017). Previously, studies on predictors of response to rTMS treatment have detected several markers such as demographic (Fregni et al., 2006; Aguirre et al., 2011; Pallanti et al., 2012; Kedzior et al., 2014), clinical (Brakemeier et al., 2008; Grammer et al., 2015; Fitzgerald et al., 2016; Rostami et al., 2017; Trevizol et al., 2020), electrophysiological (Arns et al., 2010; Narushima et al., 2010; Micoulaud-Franchi et al., 2012; Kazemi et al., 2016, 2018), and neuroimaging (Avissar et al., 2017; Corlier et al., 2019; Williams et al., 2021).

Although cognitive functions have been considered as predictors of response in the context of other interventions (Park et al., 2018) its potential as a predictor of response to rTMS, to the best of our knowledge, have thus far been considered by only two studies (Furtado et al., 2012; Hoy et al., 2012); identifying visuo-spatial working memory as predictor (Hoy et al., 2012). Apart from the investigations which have reported significant modulations of rTMS on cognitive functions in healthy populations (Kazemi et al., 2020; Patel et al., 2020; Rostami et al., 2021), various studies have considered cognitive functions as predictors of response to antidepressants. For example, early changes in facial emotion recognition has been identified as a predictor of response to antidepressants in a systematic review (Park et al., 2018). Furthermore, performance level in a verbal memory task has also been proposed as a cognitive marker (Spronk et al., 2011). However, the results are heterogeneous and some studies did not report a significant relationship between cognitive functions and treatment response (Groot et al., 1996; Doraiswamy et al., 2003; Alexopoulos et al., 2007; Lin et al., 2014; Bingham et al., 2015), and others did (Potter et al., 2004; Story et al., 2008; Bruder et al., 2014). Nevertheless, executive functions are recognized as a potential cognitive marker in most studies (Groves et al., 2018). Given that some cognitive functions have already been identified as predictors of treatment response to other modalities of interventions (Park et al., 2018) to, there is a rationale to investigate the possibility of considering them as a potential cognitive predictor of rTMS treatment response. Thus a group of cognitive functions, commonly called *cold cognition*, referring specifically to an absence of any emotional influence during the processing of the information, i.e., with emotionally neutral stimuli and without motivational relevance (Roiser and Sahakian, 2013).

In the study of Hoy et al. (2012), subjects received four different TMS protocols collectively. However, since the rTMS target region can significantly impact the outcome in question, focusing on a single protocol seems necessary to reliably draw conclusions on the involvement of a specific brain region in the variable of interest. Although a few studies have investigated the efficacy of rTMS to improve cognitive functioning BDD patients (Hu et al., 2016; Myczkowski et al., 2018; Yang et al., 2019), one aim of the current work has been on the potential of baseline cognitive functioning to predict the response to rTMS treatment in both BDD and UDD patients, which has been less explored in general, even less in the context of rTMS treatment, and least in the context of UDD and BDD. Therefore, using a bilateral rTMS protocol, we aimed to (1) examine and compare rTMS efficacy in two groups of patients (BDD & UDD) and (2) identify cognitive markers of treatment response to rTMS in the two subgroups and in all the depressed patients as a whole.

## Materials and Methods

### Participants

In total, out of 135 MDD patients (71 female), 120 (Mean age = 33.58) who received rTMS treatment in Atieh Clinical Neuroscience Center, Tehran, Iran, were analyzed (56 UDD and 64 BDD), in a retrospective naturalistic investigation. The diagnosis of depression was made by a psychiatrist according to DSM-V criteria through clinical interviews. Patients with BDI-II scores equal and above 18 were included in the study. All patients had received 20 sessions of rTMS treatment and provided informed written consent forms.

### rTMS Treatment Parameters

The 20 sessions of rTMS was performed by a Neuro MS stimulator with a 70 mm, air-cooled figure of 8 coil (Neurosoft, Russia). A bilateral stimulation protocol was used for all the patients. In every session, rTMS was applied on the right DLPFC and the left DLPFC. Resting motor threshold (RMT) was defined as the lowest stimulation intensity required for a visible muscle reflex in Abductor Policies Brevis (APB) after a minimum of 5 out of 10 single TMS pulses. The 10-HZ rTMS protocol on left DLPFC consisted of 75 trains each of which lasting for 5 s, inter-train intervals of 10 s, and stimulation intensity of 110% of the individual RMT, meaning 3,750 pulses in each session (75,000 total pulses during the course of treatment). The right DLPFC stimulation protocol however included 1 Hz 10 s trains, with an inter-train interval of 2 s. A total of 150 trains were delivered at an intensity of 120% of individual RMT, making it 1,500 pulses in each treatment session (30,000 pulses in total).

### Outcome Measures

The Beck Depression Inventory—Second Edition (BDI-II) was used to assess the primary outcomes. The criterion for response to treatment was considered minimum 50 percent decrease in BDI-II scores after the end of the treatment course, and remission was defined as BDI-II scores of <8 at the end of the treatment course. Cambridge Neuropsychological Test Automated Battery (CANTAB®, Cambridge Cognition Ltd., United Kingdom) has been applied for cognitive assessments. More details regarding CANTAB tasks and specifications can be found in the literature (Lawrence et al., 1996), however, a brief description will follow.

#### Rapid Visual Information Processing

RVIP is a visual task used for evaluating sustained attention (Hilti et al., 2010). The variables in RVIP were as follows: (1) A′ which quantifies the subject's tendency to respond, (2) B" which is a measure of the response tendency of the subject, (3) mean latency which is simply the average time it took the participant to respond correctly, (4) probability of hits which is calculated by dividing total hits over total misses plus total hits, (5) total correct rejections being the number of times when the examinee does not respond when they are not supposed to respond, and (6) total hits which is simply the number of correct responses.

#### Spatial Working Memory

SWM is a self-ordered search task evaluating non-verbal working memory (Owen et al., 1990, 1995). There are three types of errors in this task: (I) Within errors (Searching a box that has already been found to be empty; (II) between errors (revisiting a box where a token had previously been found); and (III) Double errors that contain both a within error and between error. There are two significant indices: (1) strategy utilization which is the number of search sequences starting with a novel box in both 6- and 8-box problems, and (2) errors in total, which is calculated based on the between errors, within errors, and double errors of particular box problems (i.e., between errors + within errors–double errors). This test evaluates working memory and utilization of search strategies and is a relatively accurate tool for measuring working memory, frontal lobe function, and executive functions (Oi et al., 2017; Beattie et al., 2018).

#### Delayed Matching to Sample

This task evaluates the capacity to identify complicated visual patterns after different durations of delay between stimuli (e.g., 0, 4, or 12 s) (Cambridge Cognition Ltd., 2012; Toornstra et al., 2020). In another version of the DMS test, participants are required to respond to one of 1, 2, 4, or 8 peripheral shapes that match the one present on the center of the screen simultaneously. To perform a recognition memory test, participants must memorize a visual pattern and then determine which of the four presented patterns is identical to the memorized one (the delay between the target stimulus and response differ between 0, 4, 8, and 12 s).

#### One Touch Stockings of Cambridge

OTS is an executive function test to assess spatial planning and working memory, developed based on the Tower of London test (Shallice, 1982; Sahakian et al., 1988; Owen et al., 1990). In this test, the aim is to determine how many moves are required to make a display look like the other (each moved ball equals one move). The outcome measures include “Problems solved on first choice,” “Mean choices to correct,” “Mean latency (speed of response) to first choice,” and “Mean latency to correct.” Each of these measures may be calculated for all problems or problems with a specified number of moves (1-move to 5 or 6).

### Statistical Analysis

All data analyses were performed using the SPSS software, version 19.0 (IBM, SPSS, Inc., Chicago, IL), with *p*-values below 0.05 as statistically significant. Considering the fact that no outliers were present in the data and based on relevant statistical tests, data distribution was considered normal thus parametric tests were used all throughout the analyses. To describe CANTAB scores, means ± SD were reported. To investigate the overall efficacy of rTMS treatment and pre and post-treatment differences (before and after 20 rTMS sessions), paired *t*-tests were first used for all patients irrespective of their specific diagnosis, and then used separately for UDD and BDD patients. Furthermore, a marginal model using generalized estimating equations (GEE) was used to investigate the effect of depression type (i.e., UDD and BDD) on performance in CANTAB tasks (model 1; 2 groups). A second marginal model using GEE was also used to test the effect of depression types on cognitive functions as a result of the intervention, by considering treatment response or remission (model 2; 4 groups, i.e., UDD responders, UDD non-responders, BDD responders, BDD non-responders, UDD remitters, UDD non-remitters, BDD remitters, and BDD non-remitters). All post versus pre comparisons were performed separately in each test for each variable in order to avoid the problem of multiple comparisons. For example, A′ scores of RVP have been considered as the response variable and the effect of time (pre and post treatment) have been investigated in the form of a covariate by selecting the independent working correlation matrix and a linear model in the in the GEE analysis.

Next, to investigate potential demographic and/or cognitive rTMS response predictors in responded vs. non-responded and remitted vs. non-remitted patients among all patients, UDD patients, and BDD patients, binary logistic regressions with a stepwise backward selection of variables were performed. Demographic variables, namely age and sex, and the related CANTAB test scores were considered as independent variables. A binary treatment response classification was considered as outcome meaning that “responder” and “non-responder” data were represented by one and zero. Similarly, a binary response classification was used as an outcome variable meaning that the data associated with “remitted” vs. “non-remitted” groups as one and zero, respectively. Due to internal correlations among test scores, separate regression models were used for each CANTAB test. Also, response and remission values were used as dependent variables separately in each model for every individual CANTAB test.

## Results

Two Supplementary Materials containing figures and statistical values regarding all the cognitive assessments are provided in the supplementary section of the paper.

### Data Overview

From the original sample of patients, 15 cases were excluded because they had not completed 20 sessions of rTMS treatment, thus 120 patients (age: 33.58 ± 11.1) were considered for further analysis, consisting of 71 women (age: 33.18 ± 10.19) and 49 men (age: 34.16 ± 12.31). Out of the 120 patients, 56 had a diagnosis of UDD and 64 of BDD. The treatment was well-tolerated in all patients. Aside from a mild headache after the rTMS session, usually disappearing within 1–2 days after, no adverse effects were reported. After the completion of the treatment course, 64 patients (53%) experienced at least 50% reduction in depressive symptoms (responded), and 41 patients (34%) achieved remission (Supplementary Table 1). More specifically, of all patients who responded, 34 were UDD (53.1%), and 30 (46.9%) were BDD, and of all patients who experienced remission, 22 (53.7%) were UDD, and 19 (46.3%) were BDD. The mean and standard deviation of pre and post treatment CANTAB scores are presented in Supplementary Table 1.

### Effectiveness of rTMS Treatment

#### Overall Efficacy

The first aim was to see if there were differences between pre and post stimulation performance in RVP, SWM, DMS, and OTS. The results showed a significant increase in RVP subscales including “A′,” “B”,” “probability of hit,” “Total correct rejection,” and “Total hits” if all patients were considered as one group. Moreover, an overall significant decrease in “Mean latency” was observed in the combined group and UDD patients. Furthermore, a significant decrease in the “Probability of false alarms” and “Total false alarms” was also observed. Among all the SWM subscales, “Within-errors,” “Between-errors,” “Total errors,” “First response time (4 boxes),” “Last response time (4 boxes),” “Last response time (6 boxes),” and “Last response time (8 boxes)” significantly decreased after treatment. In DSM subscales, only “Mean correct latency (simultaneous)” showed significant decrease both in the whole group as well as in BDD patients. Finally, “Mean latency to correct,” “Mean choices to correct,” and “Mean latency to first choice” among OTS subscales significantly decreased and “Problems solved on first choice” showed significant increase in all study groups. The trends of changes in all the scores and subscores of the cognitive measures are presented in Supplementary Figures 1-7.

#### Depression Type

Marginal model 1 was used to compare the efficacy of rTMS in BDD and UDD patients and the results showed that among SWM measures, “Last response time (8 boxes)” was ~51% higher in BDD compared to UDD patients (Table 1).

**Table 1:** Results of GEE analysis regarding the effects of depression types on CANTAB scores.

	**BDD/UDD**	**β**	**SE**	**Chi^**2**^**	***P***
SWM	Between errors (working memory)	5.29	3.10	2.91	0.088
	Between errors (8 boxes)	5.02	2.72	3.39	0.065
	Within errors (4 boxes)	0.23	0.14	2.84	0.092
	Working memory & short-term memory (8 boxes)	0.96	0.58	2.76	0.097
	Total errors (8 boxes)	5.44	2.84	3.67	0.055
	Last response time (8 boxes)	4,479.05	2,258.50	3.93	**0.047**

*SWM, Spatial working memory; UDD, Unipolar depressive disorder; BDD, Bipolar depressive disorder; Significant results are in bold*.

Marginal model 2 was used to test if depression type affects performance in CANTAB scores based on response (yes, no) or remission (yes, no). The dependent variable in each model was each subset of test scores with time as a covariate in the model. Interaction of time and group can show potential differences between the effects of rTMS in UDD and BDD patients during the course of the treatment, i.e., pre and post. The significant interactions are provided in Table 1.

In marginal model 2, the interaction effects of time were calculated in UDD responded vs. BDD responded, UDD responded vs. UDD non-responded patients, and BDD responded vs. BDD non-responded patients (Tables 2–4). Among all responded patients, UDD patients showed a greater decrease in B" as well as in SWM subscales including “Between-errors (working memory)”, “Between- errors (4 boxes),” “Between-errors (8 boxes),” “Total errors (between errors + within errors—double errors),” “Total errors (4 boxes),” “Total errors (8 boxes),” and “Last response time (8 boxes)” compared to BDD patients. The data for each variable is presented in Table 2. Furthermore, UDD responded patients exhibited 89 percent decrease in “Between-errors (4 boxes),” among SWM subscales, whereas “Between-errors (6 boxes)” and “Total errors (6 boxes)” had a 99% increase in UDD non-responded patients. This indicates that UDD responded patients showed significant improvements in working memory compared to UDD non-responded patients. Lastly, BDD responded patients showed a significant increase in “Between errors (working memory),” and “Total errors (working memory + short-term memory)” compared to BDD non-responded patients. Among OTS subscales, BDD responded patients showed a significant increase in “Problems solved on first choice” compared to BDD non-responded patients, indicating that executive functions improved more in BDD responded compared to BDD non-responded patients.

**Table 2:** Results of GEE analysis regarding the effects of depression types and response to treatment on CANTAB scores (UDD responders vs. BDD responders).

	**(Yes-UDD)/(Yes-BDD)**	**β**	**SE**	**Chi^**2**^**	***P***
SWM	Between errors (working memory)	−11.52	3.81	9.13	**0.003**
	Between errors (4 boxes**)**	−2.12	0.94	5.03	**0.025**
	Between errors (8 boxes**)**	−8.07	3.82	4.46	**0.035**
	Total Errors (between errors + within errors–double errors)	−10.95	4.53	5.83	**0.016**
	Total Errors (4 boxes)	−1.18	0.52	5.18	**0.023**
	Total Errors (8 boxes)	−8.49	3.95	4.62	**0.032**
	Last response time (8 boxes)	−4,920.67	2,059.33	5.71	**0.017**
DMS	B"	−0.34	0.17	3.89	**0.049**

*SWM, Spatial working memory; UDD, Unipolar depressive disorder; BDD, Bipolar depressive disorder; Significant results are in bold*.

**Table 3:** Results of GEE analysis regarding the effects of depression types and response to treatment on CANTAB scores (UDD responders vs. UDD Non-responders).

	**(Yes-UDD)/(No-UDD)**	**β**	**SE**	**Chi^**2**^**	***P***
SWM	Between errors (4 boxes**)**	−2.25	0.96	5.51	**0.019**
	Between errors (6 boxes**)**	5.28	2.62	4.06	**0.044**
	Total errors (6 boxes)	5.69	2.73	4.34	**0.037**

*SWM, Spatial working memory; UDD, Unipolar depressive disorder; BDD, Bipolar depressive disorder; Significant results are in bold*.

**Table 4:** Results of GEE analysis regarding the effects of depression types and response to treatment on CANTAB scores (BDD responders vs. BDD Non-responders).

	**(Yes-BDD)/(No-BDD)**	**β**	**SE**	**Chi^**2**^**	***P***
SWM	Between errors (working memory)	11.43	3.81	9.02	**0.003**
	Total errors (between errors + within errors–double errors)	11.26	4.13	7.44	**0.006**
OTS	Problems solved on first choice	1.27	0.62	4.23	**0.040**

*SWM, Spatial working memory; OTS, One touch stockings of Cambridge; UDD, Unipolar depressive disorder; BDD, Bipolar depressive disorder; Significant results are in bold*.

### Predictors of rTMS Treatment

Logistic regression (Backward Wald method) was used to find predictors of treatment response or remission. Age and sex were entered into each model as covariates. First, the model was performed on all patients as a whole and then separately on UDD and BDD groups. The dependent variable was either remission or response, and CANTAB scores were considered as independent variables separately. For example, in DMS, which included 13 sub-scores thus 13 independent variables plus two covariates, namely age and sex were entered into the model. The results of this statistical model is reported in Tables 5–7.

**Table 5:** Binary logistic regression analysis: demographic and DMS predictors of response or remission.

**Dependent variable**		**DMS**	**β**	**S.E**.	**Chi^**2**^**	***p***	**Exp (B)**	**95% C.I. for Exp (β)**	
								**Lower**	**Upper**
Response	Total	Gender(Male/Female)	2.29	1.07	4.56	**0.033**	9.91	1.21	81.36
		Age	0.14	0.05	7.34	**0.007**	1.15	1.04	1.27
		B"	−7.46	2.89	6.66	**0.010**	0.00	0.00	0.17
		Mean latency	−0.00	0.00	7.27	**0.007**	1.00	1.00	1.00
		Prob error given correct	−27.93	10.67	6.85	**0.009**	0.00	0.00	0.00
	UDD	Age	0.14	0.06	6.07	**0.014**	1.15	1.03	1.28
		Mean latency (all delays)	−0.00	0.00	3.64	0.056	1.00	1.00	1.00
		Prob error given correct	−13.75	7.16	3.69	0.055	0.00	0.00	1.33
Remission	Total	Gender (Male/Female)	3.26	1.37	5.67	**0.017**	26.02	1.78	380.20
		Age	0.19	0.07	7.70	**0.006**	1.21	1.06	1.39
		B"	−14.71	5.68	6.71	**0.010**	0.00	0.00	0.03
		Mean correct latency (simultaneous)	−0.00	0.00	7.12	**0.008**	1.00	0.99	1.00
		Prob error given correct	−47.96	19.30	6.18	**0.013**	0.00	0.00	0.00
		Prob error given error	13.18	7.89	2.79	0.095	5.31E + 05	0.10	2.78E + 12
	UDD	Gender (Male/Female)	5.26	2.32	5.13	**0.024**	192.06	2.03	18,169.02
		Age	0.40	0.21	3.67	0.055	1.50	0.99	2.26
		Mean correct latency (simultaneous)	−0.01	0.00	3.93	**0.048**	0.99	0.98	1.00
		Prob error given error	−39.83	24.62	2.62	0.106	0.00	0.00	4,552.94

*DMS, Delayed matching to sample; UDD, Unipolar depressive disorder; Significant results are in bold*.

**Table 6:** Binary logistic regression analysis: demographic and SWM predictors of response or remission.

**Dependent variable**		**SWM**	**β**	**S.E**.	**Chi^**2**^**	***p***	**Exp (β)**	**95% C.I. for Exp (β)**	
								**Lower**	**Upper**
BDD	Response	Gender (Male/Female)	1.46	0.78	3.47	0.063	4.30	0.93	20.00
		Between errors (4 boxes)	−0.75	0.28	6.93	**0.008**	0.47	0.27	0.83
		Strategy utilization	0.14	0.08	2.83	0.092	1.15	0.98	1.34
		Last response time (4 boxes)	0.00	0.00	5.55	**0.019**	1.00	1.00	1.00
		Last response time (8 boxes)	0.00	0.00	2.81	0.094	1.00	1.00	1.00
	Remission	Gender (Male/Female)	1.30	0.82	2.51	0.113	3.68	0.73	18.49
		Age	−0.06	0.05	1.67	0.196	0.94	0.86	1.03
		Between errors (4 boxes)	−0.61	0.28	4.69	**0.030**	0.54	0.31	0.94
		Within errors (4 boxes)	1.30	1.47	0.78	0.376	3.68	0.21	66.00
		Within errors (6 boxes)	−0.15	0.18	0.72	0.397	0.86	0.60	1.23
		Working memory + Short-Term memory (4 boxes)	−5.92	4.83	1.50	0.221	0.00	0.00	34.89
		Strategy utilization	0.14	0.08	2.94	0.087	1.15	0.98	1.34
		First response time (4 boxes)	0.00	0.00	1.94	0.164	1.00	0.99	1.00
		First response time (6 boxes)	0.00	0.00	2.29	0.130	1.00	1.00	1.00
		Last response time (4 boxes)	0.00	0.00	4.12	**0.042**	1.00	1.00	1.00
		Last response time (8 boxes)	0.00	0.00	2.27	0.132	1.00	1.00	1.00

*SWM, Spatial working memory; BDD, Bipolar depressive disorder; Significant results are in bold*.

**Table 7:** Binary logistic regression analysis: demographic and OTS predictors of remission.

**Dependent variable**		**OTS**	**β**	**S.E**.	**Chi^**2**^**	***P***	**Exp (β)**	**95% C.I. for Exp (β)**	
								**Lower**	**Upper**
Remission	Total	Gender (Male/Female)	0.85	0.44	3.79	0.052	2.34	0.99	5.51
		Mean choices to correct	−3.49	1.38	6.41	**0.011**	0.03	0.00	0.45
		Mean latency to correct	0.00	0.00	8.50	**0.004**	1.00	1.00	1.00
		Mean latency to first choice	0.00	0.00	8.62	**0.003**	1.00	1.00	1.00
	UDD	Age	0.08	0.03	5.54	**0.019**	1.08	1.01	1.16
		Mean choices to correct	−6.23	2.61	5.70	**0.017**	0.00	0.00	0.33
		Mean latency to correct	0.00	0.00	5.14	**0.023**	1.00	1.00	1.00
		Mean latency to first choice	0.00	0.00	6.06	**0.014**	1.00	1.00	1.00

*OTS, One touch stockings; UDD, Unipolar depressive disorder; Significant results are in bold*.

For DMS scores, when response was used as an independent variable, the odds of the “B” (β = −7.5, *p* ~ 0.010), “Mean latency” (β = −0.003, *p* ~ 0.007), and “Prob error given correct” (β = −27.9, *p* ~ 0.009) were lower among responded compared to non-responded patients as a whole (i.e., without dividing them into BDD and UDD).Also, males were 9.91 times more likely to respond than females. If age increases by one, the odds ratio increases by 15 percent. In addition, we observed that in UDD patients, the odds ratio of “Mean latency (all delays) ” (β = −0.002, *p* ~ 0.056), and “Prob error given correct” (β = −13.7, *p* ~ 0.055) were lower among responded compared to non-responded patients in a marginally significant manner (*p* < 0.1). Also, if age increases by one, the odds increases by 15 percent.

For DMS scores, when remission was used as an independent variable, the odds ratio of the “B"” (β = −14.7, *p* ~ 0.010), “Mean correct latency (simultaneous)” (β = −0.005, *p* ~ 0.008) and “Prob error given correct” (β = −48.0, *p* ~ 0.013) were lower among remitted compared to non-remitted patients as a whole. Furthermore, males were 26 times more likely to reach remission compared to females. If age increases by one, the odds ratio increases by 21 percent. Moreover, in UDD patients, the odds ratio of “Mean correct latency (simultaneous)” was lower among remitted compared to non-remitted patients (β = −0.01, *p* ~ 0.048) and males were more likely to reach remission than females.

For SWM scores, when response to treatment was used as an independent variable, in BDD patient, the odds ratio of the “Between-errors (4 boxes)” (β = −0.75, *p* ~ 0.008) and “Last response time (4 boxes)” (β = 0.0003, *p* ~ 0.019) were lower among responded compared to non-responded patients. In BDD patients, when considering SWM scores with remission as the independent variable, the odds ratio of the “Between-errors (4 boxes)” (β = −0.61, *p* ~ 0.030) and “Last response time” (β = −0.0004, *p* ~ 0.042) were lower among remitted compared to non-remitted patients.

Considering OTS scores with treatment remission as the independent variable, the odds of the “Mean choices to correct” (β = −3.5, *p* ~ 0.011), “Mean latency to correct” (β = 0.0007, *p* ~ 0.004), and “Mean latency to first choice” (β = −0.0008, *p* ~ 0.003) were lower among remitted compared to non-remitted patients as a whole. Lastly, in UDD patients, the odds of the “Mean choices to correct” (β = −6.23, *p* ~ 0.017), “Mean latency to correct” (β = 0.001, *p* ~ 0.023), and “Mean latency to first choice” (−0.001, *p* ~ 0.014) were lower among remitted compared to non-remitted patients. If age increases by one, the odds ratio increases by 8%.

## Discussion

The results of this study showed no significant difference in the efficacy of bilateral rTMS on cognitive function in unipolar and bipolar depression. Considering response rates (responded vs. non-responded), the efficacy of rTMS in improving working memory in UDD and BDD patients was significantly different. Considering remission rates (remitted vs. non-remitted), the efficacy of rTMS in improving working memory and visual memory was different among UDD and BDD patients as well. Cognitive predictors of response and remission in both patient groups (UDD and BDD) were determined. Taking all the patients as a whole, visual memory, age, and sex were determined as the predictors of response to rTMS. However, visual memory and age in UDD patients and working memory performance in BDD patients were identified as predictors of treatment response. Regarding remission, when considering all patients as one group, visual memory, age, sex, and executive functions were identified as remission predictors. Executive functions and age; and working memory were identified as remission predictors in UDD and BDD patients, respectively.

### rTMS Efficacy on Cognitive Functions

#### rTMS Outcome Regarding Cognitive Functioning of UDD and BDD Patients

Our findings showed that bilateral rTMS improved cognitive functioning in general and sustained attention, working memory, and executive functions in particular, in depressed patients. Taking depression types into account, a significant difference was observed between the two types of depression in working memory performance, before and after treatment. UDD patients showed more improvement in working memory than BDD patients, but no significant difference was observed in other cognitive functions. It should be noted that in the absence of a more sophisticated control condition, it would not be possible to dissociate the effect of the improvement in cognitive function on mood, or vice versa thus reliably concluding that one is the cause and the other the effect.

However, previous studies which investigated efficacy of rTMS in improving cognitive functions in both clinical and healthy population report seemingly diverging results. A meta-analysis including 18 studies, on the effects of rTMS on cognitive functions in MDD patients reported no significant effect on cognitive functions (Martin et al., 2017) in accordance with another meta-analysis on healthy population (Patel et al., 2020). However, some promising results have also been observed (Patel et al., 2020) suggesting that high-frequency rTMS of DLPFC could improve executive functioning, and low-frequency rTMS could enhance episodic memory and visual perception. However, another meta-analysis showed that rTMS improved working memory in both healthy and clinical populations in all indexes and tDCS only improved reaction time (Brunoni and Vanderhasselt, 2014).

Similar to the context of rTMS effects on clinical symptoms, it is possible to attribute the variability in rTMS outcomes regarding cognitive symptoms to non-homogeneity in stimulation parameters in different studies since for example, frequency of stimulation has been shown to play a significant role (Patel et al., 2020). Thus, it is safe to assume that other stimulation parameters such as the total number of pulses or the intensity of the stimulation can potentially impact the rTMS outcome. Furthermore, number of pulses in each session and total number of sessions are other points of difference with similar studies in this context, that may explain our different results (Koren et al., 2001; Mottaghy et al., 2002; Rami et al., 2003; Huang et al., 2004; Vanderhasselt et al., 2007; Viggiano et al., 2008; Barr et al., 2009; Upton et al., 2010; Kim et al., 2012; Gaudeau-Bosma et al., 2013; Fried et al., 2014; Pearce et al., 2014). In the current study, a relatively higher number of pulses were administered compared to the studies included in both of the meta-analyses mentioned previously and it is noteworthy that a previous study on the effects of different durations of rTMS on cortical inhibition and excitability showed that longer sessions with a greater number of pulses could result in more pronounced changes in cortical inhibition followed by rTMS (de Jesus et al., 2014), which can significantly contribute to a significant modulation in the behavior or cognitive function in question. It seems that concerns regarding rTMS safety parameters generally lead many researchers to use protocols with minimum suggested values in each stimulation parameter (e.g., minimum number of total pulses per session, minimum number of sessions, and longer inter-train intervals) although the latest clinical safety guideline emphasizes that with a proper use of RMT, it is not necessary to use the suggested parameters in the previous two guidelines (Rossi et al., 2021). Furthermore, there is also evidence regarding the possibility of using shorter inter-train intervals (Cash et al., 2017) which is also a potential candidate to maximize the possibility of observing an outcome. There are studies that have corroborated this finding in different clinical population (e.g., stroke) (Ke et al., 2020) and also the results of two of our previous investigations using this protocol revealed a very acceptable response rate (Kazemi et al., 2016, 2018). The stimulation intensity is another parameter that has a decisive role in the response rate to rTMS in patients with depression (Fitzgerald et al., 2016) and it has been shown that higher stimulation intensities are associated with better treatment response (Padberg et al., 2002; Fitzgerald et al., 2016). Altogether, the use of a higher stimulation intensity and higher number of pulses in the current study could have contributed to observed effects of rTMS effects on cognitive function.

#### rTMS Outcome Regarding Cognitive Functioning in Responders vs. Non-responders

In general, responded patients showed more improvements in cognitive functioning than non-responded patients. In particular, more improvements were observed in working memory performance in responded BDD compared to responded UDD patients (i.e., bipolar responded vs. unipolar responded). These results are in line with previous studies (Bailey et al., 2018) according to which, MDD patients who responded to treatment showed significant improvements in working memory compared to non-responding patients (Bailey et al., 2018). There is evidence regarding more pronounced reduction in DLPFC activity in MDD compared to BDD patients while performing a working memory task (Zhu et al., 2018; Manelis et al., 2020) which can be one of the reasons behind the finding that BDD patients showed more improvements in working memory in our study. Moreover, there are reports showing that UDD patients exhibit more gray matter reduction in DLPFC in both hemispheres compared to BDD patients (de Azevedo-Marques Périco et al., 2011) thus possibly contributing to the relatively less improvement in working memory in UDD patients observed in this study. This pattern seems consistent with the findings of another study in which BDD patients showed more improvements in working memory compared to UDD patients (Xu et al., 2012).

### Predictors of Treatment Response and Remission

#### Treatment Response Prediction in UDD and BDD Patients

Compared to studies using hot cognition as predictor of treatment response to anti-depressants, fewer studies have focused on cold cognition (~26 vs. 3 papers) (Seeberg et al., 2018). In the context of rTMS treatment, to the best of our knowledge, three studies focused on cognitive predictors (hot cognition), among which one has only used cognitive assessments (Hoy et al., 2012) and others also took advantage of the fMRI methodology (Furtado et al., 2012; Hernández-Ribas et al., 2013). In the first study, involving 137 participants, immediate visual-spatial memory was considered as predictor of response to rTMS (Hoy et al., 2012). The second study found that while performing an executive function task (i.e., word generation), responders show lower activity in perigenual, medial OFC and middle frontal cortices as well as higher activity in ventral- caudal putamen in baseline (Hernández-Ribas et al., 2013). Finally, the third study found no significant differences between responders and non-responders in cognitive performance. All of these studies were performed on MDD patients, but in the study of Hoy et al., 4 out of 137 and in the study of Furtado et al., 6 out of 21 patients were diagnosed with bipolar disorder. Furthermore, different numbers of protocols were used in each study (Furtado et al., 2012; Hoy et al., 2012; Hernández-Ribas et al., 2013). Regardless of depression types, we found visual memory, age, and sex to act as predictors of response to rTMS. This is in line with the previous findings using cognitive performance measures (Hoy et al., 2012) and a study used combined approach (Hernández-Ribas et al., 2013).

When taking depression types into account, we observed some differences that previous studies have not reported, possibly due to smaller sample sizes. While there is evidence that BDD patients have difficulties in both cold and hot cognition (Roiser et al., 2009), to the best of our knowledge, most studies have focused on cold cognition in UDD patients, and thus far, there has been no study on BDD patients in that respect (Seeberg et al., 2018). The results of the current investigation showed that working memory; and visual memory and age could be used as predictors of response to rTMS in patients with BDD and UDD, respectively.

#### Visual Memory

In general, we found that older men with better visual memory performance responded better to treatment. Also, individuals with UDD with a better performance in visual memory tasks showed better response to the treatment. Furthermore, older men who had better scores in visual memory tests at baseline were more likely to reach remission. By older age, middle-age range and not elderly is meant. However, it is noteworthy that the older the age, the more critical the role of visual memory would be in predicting treatment response. Based on the neuroimaging evidence, different areas of the brain are activated in older vs. younger subjects while performing visual memory tasks (Bennett et al., 2001). Although the DLPFC and other frontal areas are not among the above mentioned brain regions, there is evidence showing that older adults recruit brain areas including the DLPFC and middle temporal areas while performing visual memory tasks (McIntosh et al., 1999). Therefore, considering the normal activity of these areas in older adults, the effect of rTMS on bilateral DLPFC can potentially be an indicator for predicting response to rTMS treatment.

Consistent with Hoy et al. (2012), our study revealed that visual memory could be considered as a predictor of response to rTMS (Hoy et al., 2012). Moreover, our results share similarities with another pharmacological study (Herrera-Guzmán et al., 2008) although in general visual memory has not been replicated as a response predictor to the extent that working memory and executive functions, in pharmacological studies. In our study, response to bilateral rTMS was associated with visual memory, age and sex. Age (Malik et al., 2016; Rostami et al., 2017) and sex (Huang et al., 2008; Malik et al., 2016) have previously been recognized as demographic response predictors. On the other hand, age and sex are two potential factors affecting memory performance, and in particular, visual memory performance (Pauls et al., 2013; Piccardi et al., 2016; Garg et al., 2017; Voyer et al., 2017).

#### Working Memory and Executive Function

When all the patients are considered, individuals with a better performance in executive functions at the baseline were more likely to experience remission. Among BDD patients, individuals who had better working memory performance responded better to treatment and reached remission, and in UDD patients, individuals who had better performances in executive functions at the baseline had a better chance for remission.

In line with Hernández-Ribas et al. our study also showed executive functions as a potential candidate for response prediction (Hernández-Ribas et al., 2013). Moreover, our findings are consistent with pharmacological (Dunkin et al., 2000; Alexopoulos et al., 2005; Sneed et al., 2007; Herrera-Guzmán et al., 2008; Shiroma et al., 2014; Etkin et al., 2015; Murrough et al., 2015; Bastos et al., 2017) and psychological studies (Beaudreau et al., 2015; Kundermann et al., 2015; Morimoto et al., 2016; Bastos et al., 2017). Executive functions have been recognized as one of the most important predictors of response to anti-depressants (Groves et al., 2018).

Other than pharmacological studies, few studies investigated cognitive predictors of response to psychotherapy and better executive function was associated with better response to CBT (Kundermann et al., 2015). In addition, in cognitive remediation, executive dysfunction has been shown to be associated with predicting treatment response (Morimoto et al., 2016). Discrepancies in these findings might be due to the different nature of psychological therapies. In general, regarding executive function, similar to cognitive markers of visual memory, better test performance is an indicator of normal activity in prefrontal brain regions, which is a predictor of favorable response to treatment.

## Limitations

The reported results in the current study should be cautiously interpreted as this has been a naturalistic retrospective study which means that although it mimics the real life and treatment course of patients without the active interference of researchers thus producing relatively more ecologically valid results, however, patient activities are not standardized and do not strictly follow a specific study protocol, which may introduce confounding variables affecting the results. The absence of a sham group in this study, as dictated by its design can also limit the extent to which the results can be generalized and more reliably interpreted.

## Conclusion

The cognitive neuropsychologic model for depression (Roiser et al., 2012) implies that negative emotional processing biases depend on both bottom- up responses to salient emotional stimulus and weak top-down cognitive control mechanisms which are required for responding to task-irrelevant emotional information and as decreased response of lateral frontal and dorsal ACC in situations where the response to task-irrelevant emotional information must be suppressed indicates better cognitive control and reflects normal brain activity and better adaptability, rTMS treatment outcome on brain regions involved in cognitive control can result in altered cold cognition and subsequently reduce negative biases impacting depression symptoms. In this study, cognitive predictors of response to bilateral rTMS in patients with unipolar and bipolar depression were identified. Considering their relative accessibility, using cognitive tests at baseline and before the start of the treatment can be a useful and informative approach for the prediction of treatment response and pave the way toward a more personalized and effective brain stimulation treatment.

## Data Availability Statement

The raw data supporting the conclusions of this article will be made available by the authors, without undue reservation.

## Ethics Statement

Ethical review and approval was not required for the study on human participants in accordance with the local legislation and institutional requirements. Written informed consent to participate in this study was provided by the participants' legal guardian/next of kin.

## Author Contributions

RR and RK: designed the study. AH, SA, and RK: carried out the study. ZN: analyzed the data. RR, RK, AH, and SA: interpreted the results and wrote the manuscript. RR, RK, and NJ: reviewed the final draft. All authors read and approved the final manuscript.

## Conflict of Interest

The authors declare that the research was conducted in the absence of any commercial or financial relationships that could be construed as a potential conflict of interest.

## Publisher's Note

All claims expressed in this article are solely those of the authors and do not necessarily represent those of their affiliated organizations, or those of the publisher, the editors and the reviewers. Any product that may be evaluated in this article, or claim that may be made by its manufacturer, is not guaranteed or endorsed by the publisher.
