# Supplementary material for: Cold Cognition as Predictor of Treatment Response to rTMS; A Retrospective Study on Patients With Unipolar and Bipolar Depression
Source: Front Hum Neurosci. 2022 Jul 25;16:888472. doi: 10.3389/fnhum.2022.888472 (PMC9358278; doi:10.3389/fnhum.2022.888472)
Supplement: Supplementary file 1 [file Data_Sheet_1.docx]

aa


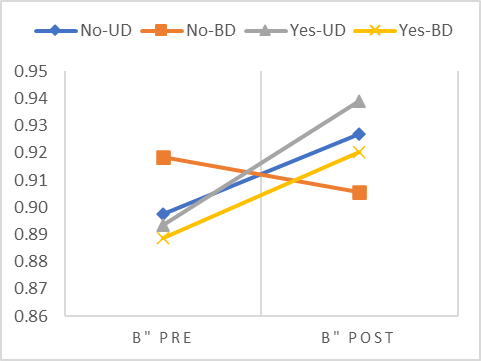

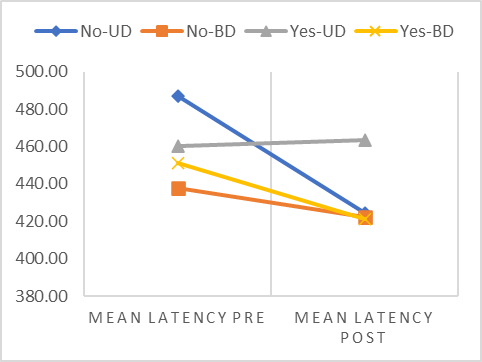

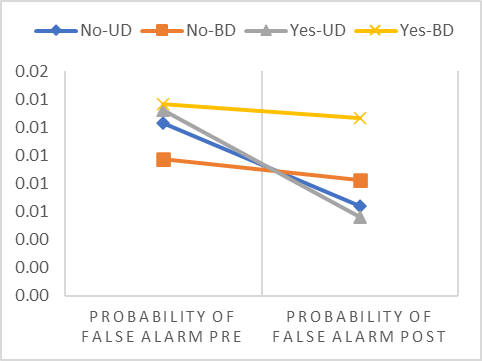

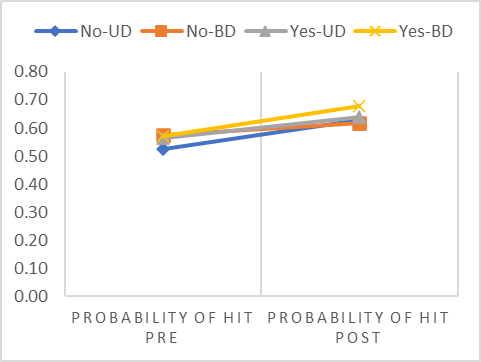

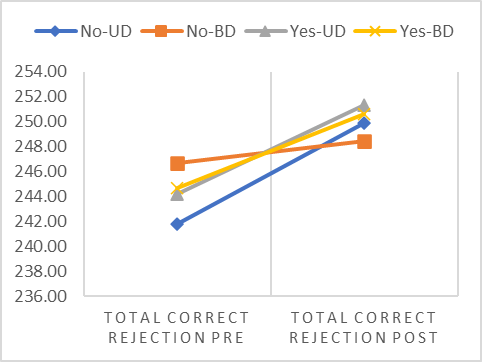

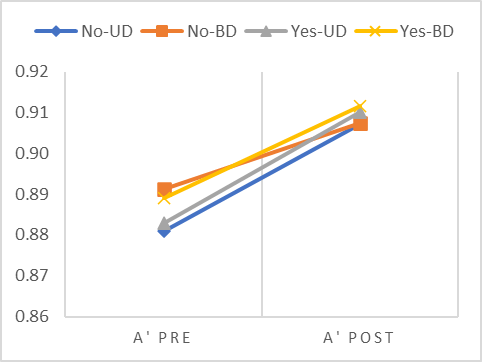

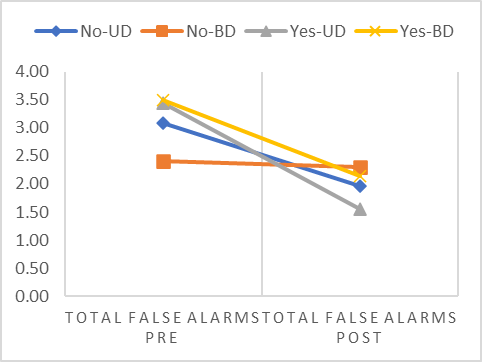

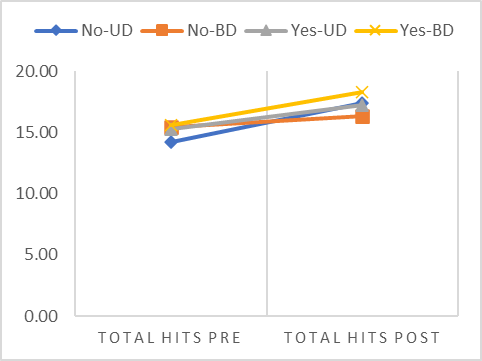

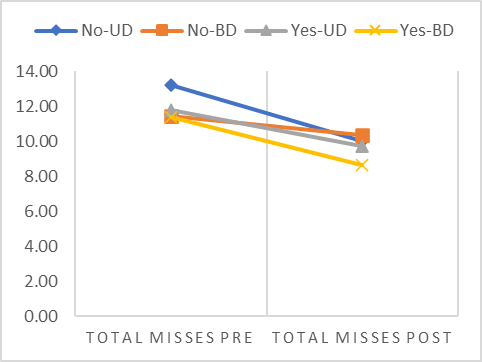


Figure 1 – Pre and post RVP scores. No-UD= Unipolar non-responders; No-BD= Bipolar non-responders; Yes-UD= Unipolar responders; Yes-BD= Bipolar responders


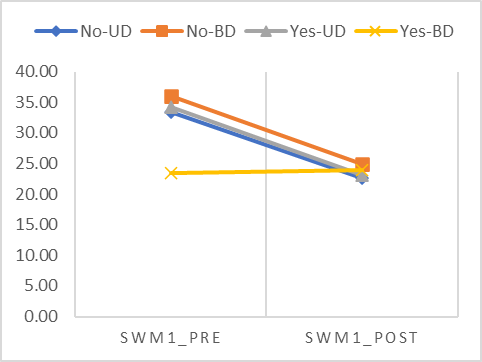

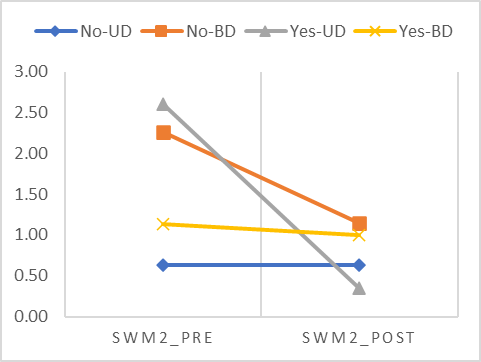

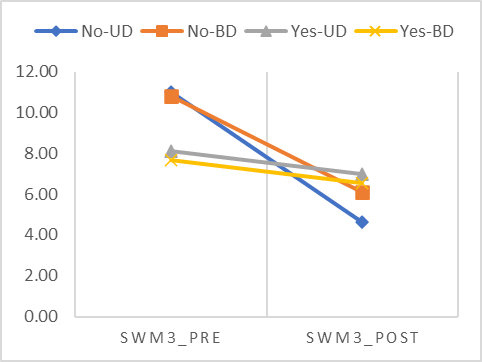

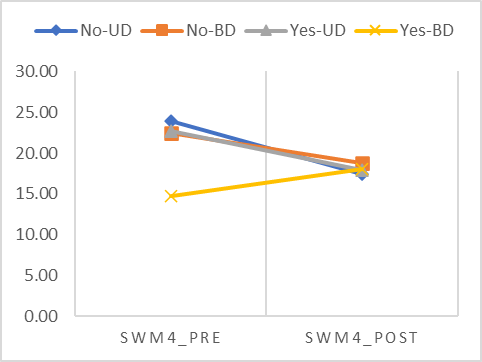

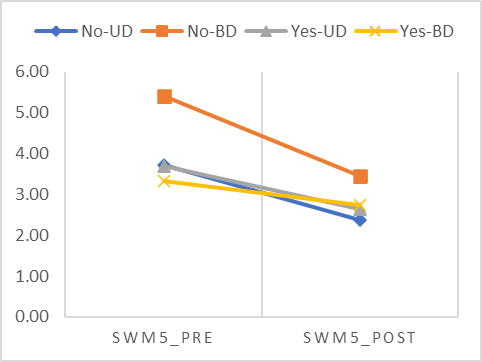

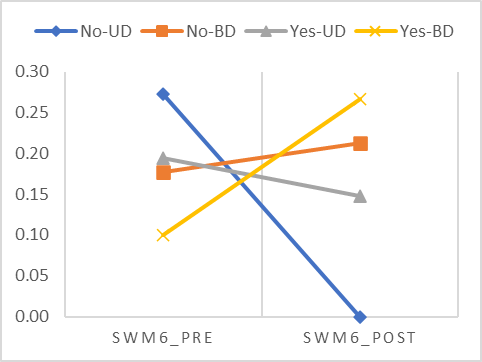

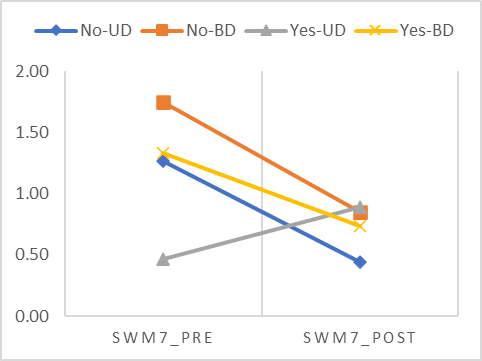

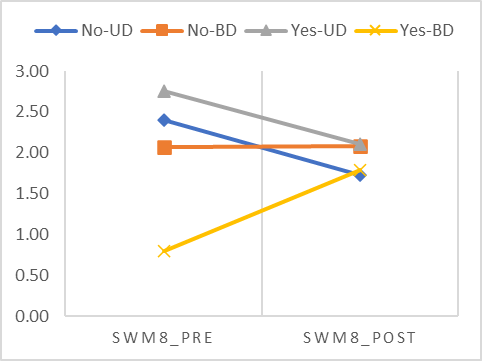

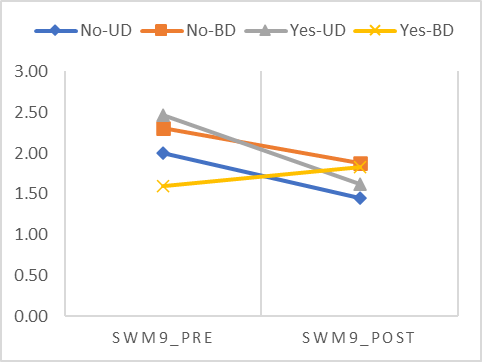


Figure 2- Pre and post SWM scores. No-UD= Unipolar non-responders; No-BD= Bipolar non-responders; Yes-UD= Unipolar responders; Yes-BD= Bipolar responders


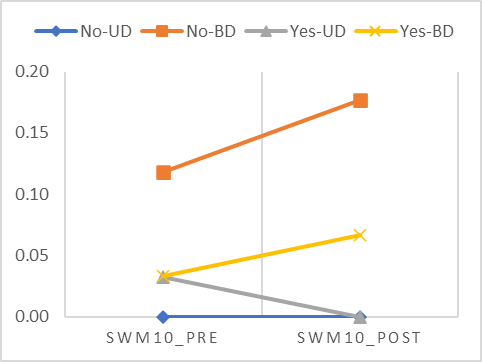

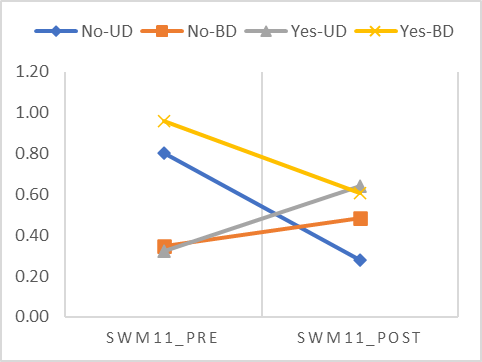

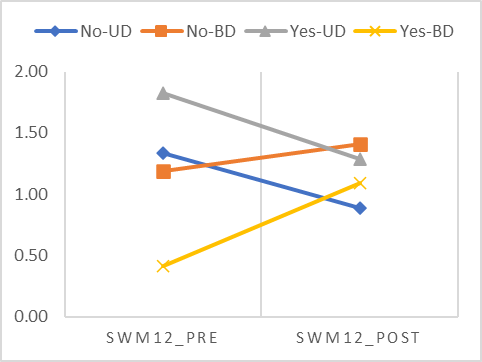

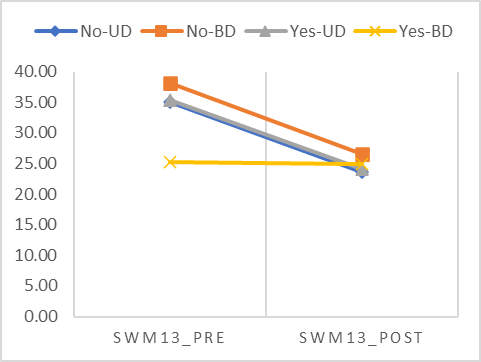

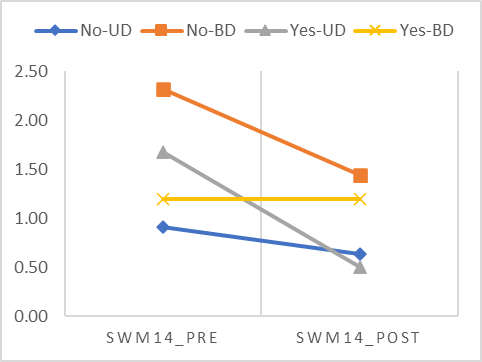

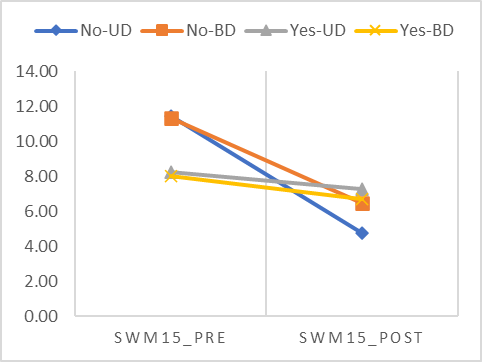

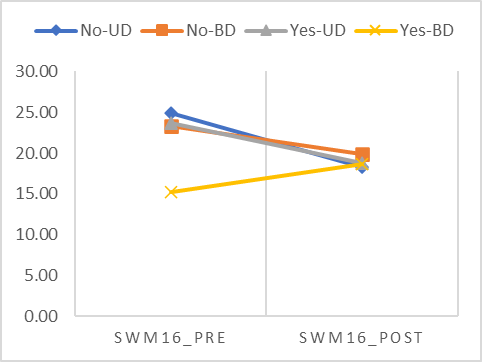

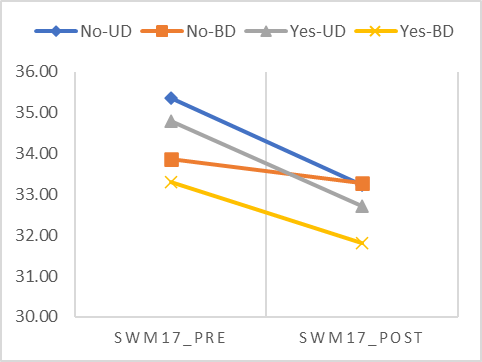

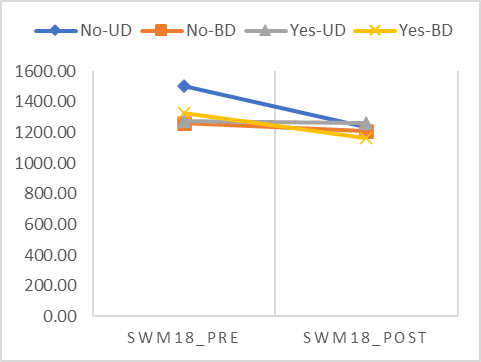


Figure 3 – Pre and post SWM scores. No-UD= Unipolar non-responders; No-BD= Bipolar non-responders; Yes-UD= Unipolar responders; Yes-BD= Bipolar responders


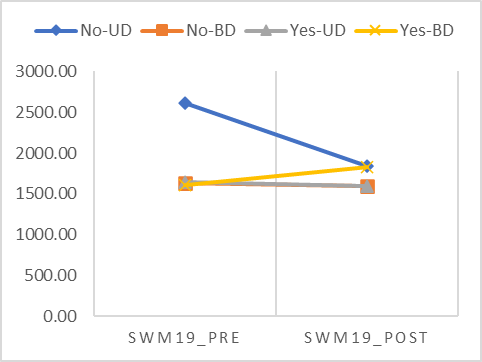

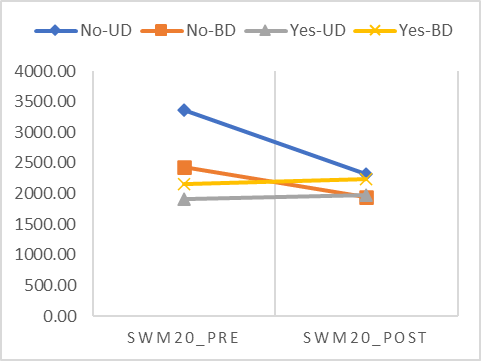

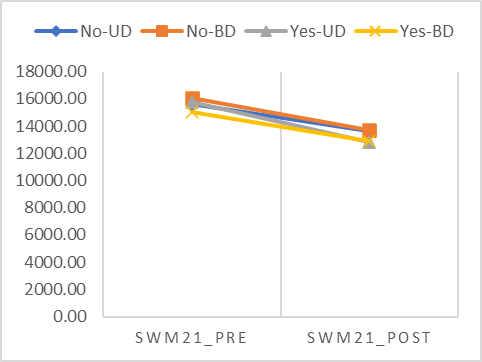

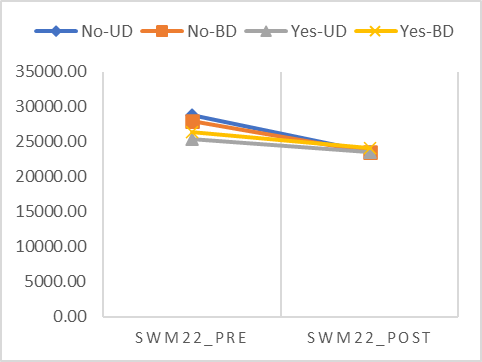

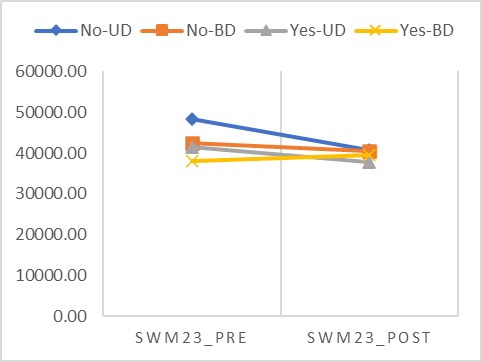


Figure 4 – Pre and post SWM scores. No-UD= Unipolar non-responders; No-BD= Bipolar non-responders; Yes-UD= Unipolar responders; Yes-BD= Bipolar responders


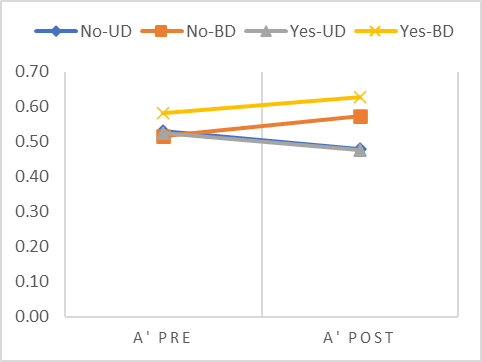

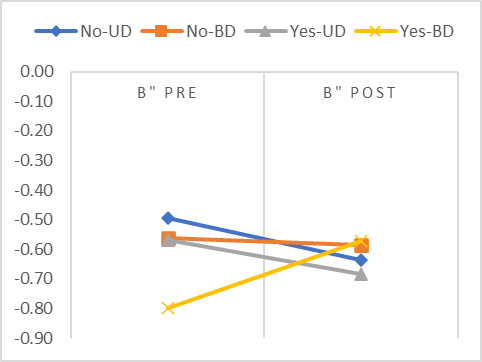

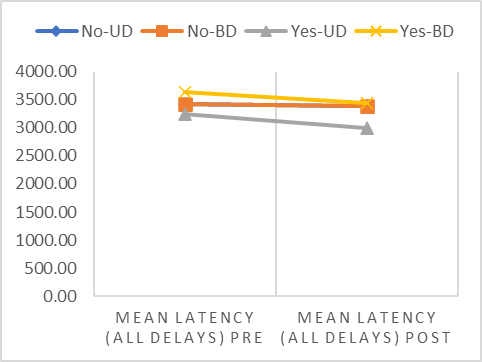

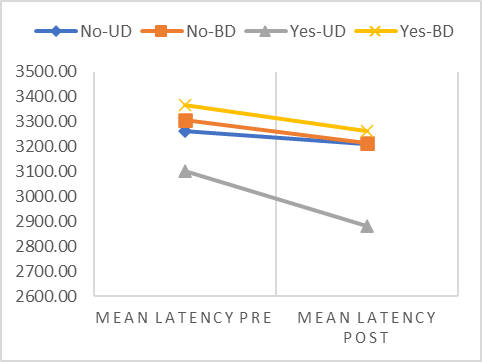

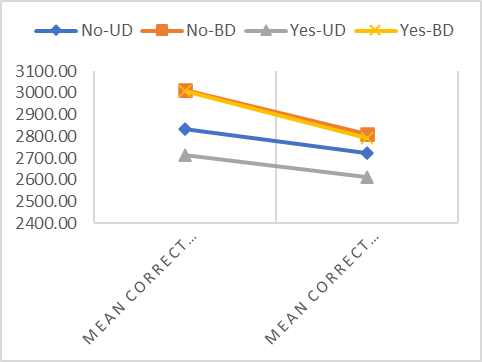

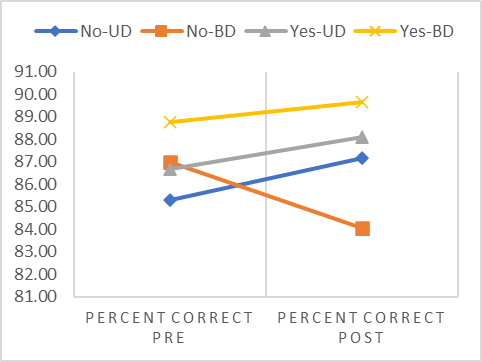

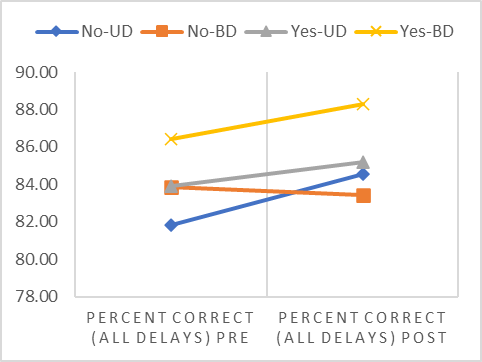

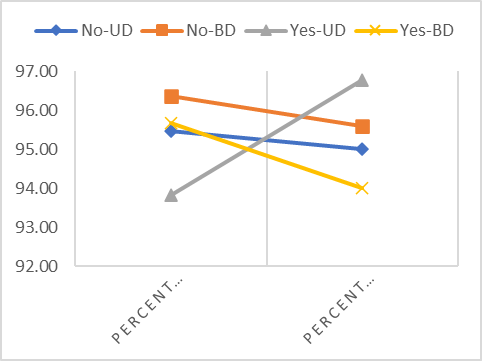

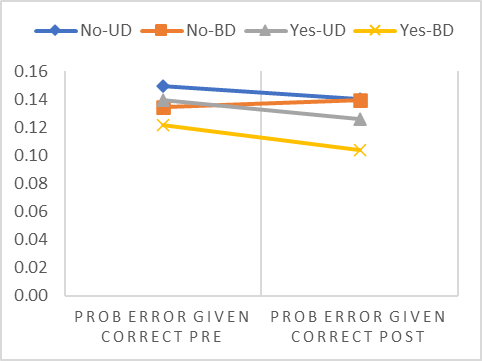


Figure 5 – Pre and post DMS scores. No-UD= Unipolar non-responders; No-BD= Bipolar non-responders; Yes-UD= Unipolar responders; Yes-BD= Bipolar responders


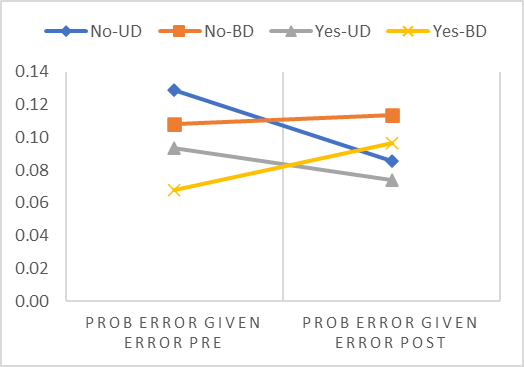

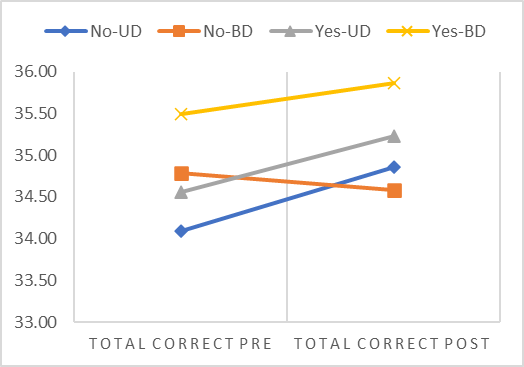

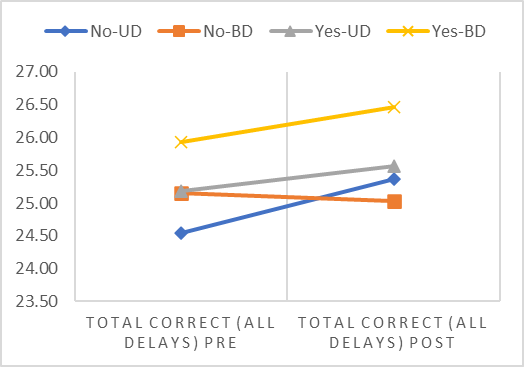

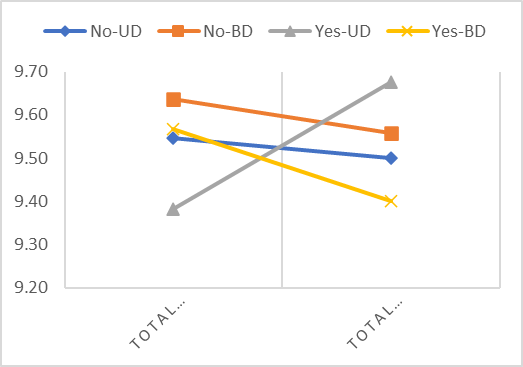


Figure 6 – Pre and post DMS scores. No-UD= Unipolar non-responders; No-BD= Bipolar non-responders; Yes-UD= Unipolar responders; Yes-BD= Bipolar responders


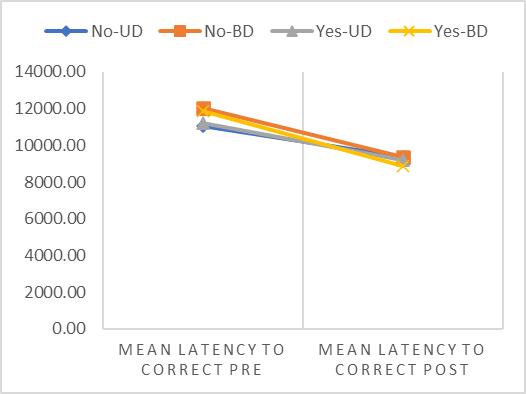

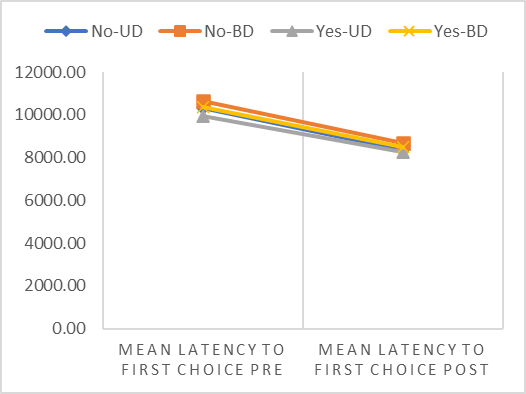

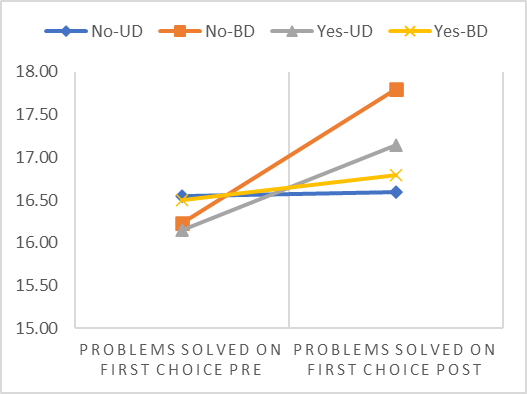

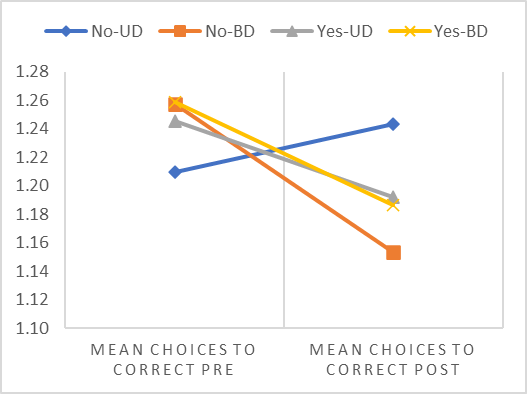


Figure 7 – Pre and post OTS scores. No-UD= Unipolar non-responders; No-BD= Bipolar non-responders; Yes-UD= Unipolar responders; Yes-BD= Bipolar responders
