# Supplementary material for: Cold Cognition as Predictor of Treatment Response to rTMS; A Retrospective Study on Patients With Unipolar and Bipolar Depression
Source: Front Hum Neurosci. 2022 Jul 25;16:888472. doi: 10.3389/fnhum.2022.888472 (PMC9358278; doi:10.3389/fnhum.2022.888472)
Supplement: Supplementary file 2 [file Table_1.docx]

Supplementary Table:

Table 1 – Results of the GEE analysis in all patient groups separately as well as combined in a single group (total), both considering response and remission, for all the variables.

Note: RVP= Rapid Visual Processing; SWM= Spatial Working Memory; DMS= Delayed Matching to Sample; OTS= One Touch Stockings of Cambridge

|  | | | Total | | | response | | | | | | remission | | | | | |
| --- | --- | --- | --- | --- | --- | --- | --- | --- | --- | --- | --- | --- | --- | --- | --- | --- | --- |
|  |  |  | Mean | SD | p | No | | P | Yes | | P | No | | P | Yes | | P |
|  |  |  |  |  |  | Mean | SD |  | Mean | SD |  | Mean | SD |  | Mean | SD |  |
| **RVP** | A' Pre | UDD | 0.88 | 0.04 | ***<0.001*** | 0.88 | 0.04 | ***0.048*** | 0.88 | 0.05 | ***0.001*** | 0.88 | 0.04 | ***0.001*** | 0.89 | 0.06 | ***0.033*** |
|  |  | BDD | 0.89 | 0.05 |  | 0.89 | 0.04 |  | 0.89 | 0.05 |  | 0.89 | 0.05 |  | 0.88 | 0.05 |  |
|  | A' Post | UDD | 0.91 | 0.05 | ***<0.001*** | 0.91 | 0.06 | 0.055 | 0.91 | 0.05 | ***<0.001*** | 0.91 | 0.05 | ***0.007*** | 0.91 | 0.05 | ***0.002*** |
|  |  | BDD | 0.91 | 0.05 |  | 0.91 | 0.05 |  | 0.91 | 0.05 |  | 0.91 | 0.05 |  | 0.91 | 0.05 |  |
|  | B" Pre | UDD | 0.90 | 0.11 | ***0.006*** | 0.90 | 0.10 | 0.110 | 0.89 | 0.13 | ***0.023*** | 0.91 | 0.09 | 0.056 | 0.88 | 0.14 | ***0.043*** |
|  |  | BDD | 0.90 | 0.11 |  | 0.92 | 0.10 |  | 0.89 | 0.13 |  | 0.92 | 0.09 |  | 0.87 | 0.15 |  |
|  | B" Post | UDD | 0.93 | 0.07 | 0.721 | 0.93 | 0.10 | 0.697 | 0.94 | 0.05 | 0.253 | 0.93 | 0.08 | 0.918 | 0.93 | 0.06 | 0.392 |
|  |  | BDD | 0.91 | 0.08 |  | 0.91 | 0.09 |  | 0.92 | 0.06 |  | 0.92 | 0.08 |  | 0.91 | 0.07 |  |
|  | Mean latency Pre | UDD | 470.43 | 110.71 | 0.173 | 486.93 | 130.26 | ***0.018*** | 460.24 | 97.43 | 0.946 | 477.14 | 115.29 | ***0.023*** | 460.37 | 105.28 | 0.731 |
|  |  | BDD | 444.00 | 109.60 |  | 437.87 | 125.95 |  | 450.96 | 89.15 |  | 440.05 | 114.64 |  | 453.64 | 98.60 |  |
|  | Mean latency Post | UDD | 447.81 | 116.19 | 0.074 | 424.28 | 99.33 | 0.328 | 463.49 | 125.19 | 0.131 | 433.00 | 97.48 | 0.129 | 470.03 | 139.19 | 0.353 |
|  |  | BDD | 421.70 | 82.04 |  | 422.32 | 75.32 |  | 421.03 | 89.95 |  | 419.22 | 70.72 |  | 427.29 | 105.31 |  |
|  | Probability of false alarm Pre | UDD | 0.01 | 0.02 | ***0.002*** | 0.01 | 0.01 | ***0.006*** | 0.01 | 0.02 | ***0.026*** | 0.01 | 0.01 | ***0.002*** | 0.02 | 0.02 | 0.060 |
|  |  | BDD | 0.01 | 0.02 |  | 0.01 | 0.02 |  | 0.01 | 0.02 |  | 0.01 | 0.02 |  | 0.02 | 0.02 |  |
|  | Probability of false alarm Post | UDD | 0.01 | 0.01 | 0.633 | 0.01 | 0.02 | 0.648 | 0.01 | 0.01 | 0.817 | 0.01 | 0.01 | 0.431 | 0.01 | 0.01 | 0.937 |
|  |  | BDD | 0.01 | 0.02 |  | 0.01 | 0.01 |  | 0.01 | 0.03 |  | 0.01 | 0.01 |  | 0.02 | 0.04 |  |
|  | Probability of hit Pre | UDD | 0.55 | 0.17 | ***0.003*** | 0.53 | 0.19 | 0.056 | 0.56 | 0.16 | ***0.029*** | 0.53 | 0.17 | ***0.024*** | 0.58 | 0.16 | ***0.027*** |
|  |  | BDD | 0.57 | 0.19 |  | 0.57 | 0.18 |  | 0.57 | 0.20 |  | 0.59 | 0.18 |  | 0.54 | 0.22 |  |
|  | Probability of hit Post | UDD | 0.64 | 0.21 | ***0.007*** | 0.63 | 0.22 | 0.315 | 0.64 | 0.20 | ***0.001*** | 0.63 | 0.23 | 0.132 | 0.64 | 0.18 | ***0.010*** |
|  |  | BDD | 0.65 | 0.20 |  | 0.62 | 0.23 |  | 0.68 | 0.16 |  | 0.63 | 0.22 |  | 0.67 | 0.15 |  |
|  | Total correct rejection Pre | UDD | 243.25 | 10.78 | ***<0.001*** | 241.82 | 10.71 | ***0.007*** | 244.18 | 10.89 | ***0.006*** | 242.29 | 9.96 | ***0.002*** | 244.73 | 12.04 | ***0.022*** |
|  |  | BDD | 245.77 | 13.55 |  | 246.71 | 14.40 |  | 244.70 | 12.67 |  | 247.11 | 13.70 |  | 242.58 | 12.98 |  |
|  | Total correct rejection Post | UDD | 250.77 | 12.55 | ***0.018*** | 249.91 | 13.79 | 0.486 | 251.32 | 11.85 | ***0.001*** | 249.79 | 13.36 | 0.192 | 252.27 | 11.30 | ***0.013*** |
|  |  | BDD | 249.48 | 12.55 |  | 248.47 | 12.48 |  | 250.63 | 12.74 |  | 249.67 | 11.94 |  | 249.05 | 14.23 |  |
|  | Total false alarms Pre | UDD | 3.30 | 4.20 | ***0.002*** | 3.09 | 3.53 | ***0.018*** | 3.44 | 4.63 | ***0.015*** | 2.82 | 3.18 | ***0.004*** | 4.05 | 5.43 | ***0.043*** |
|  |  | BDD | 2.92 | 4.21 |  | 2.41 | 3.76 |  | 3.50 | 4.67 |  | 2.36 | 3.46 |  | 4.26 | 5.49 |  |
|  | Total false alarms Post | UDD | 1.71 | 2.68 | 0.272 | 1.95 | 3.85 | 0.870 | 1.56 | 1.58 | 0.103 | 1.71 | 3.14 | 0.667 | 1.73 | 1.83 | 0.176 |
|  |  | BDD | 2.22 | 2.32 |  | 2.29 | 2.60 |  | 2.14 | 2.00 |  | 2.11 | 2.33 |  | 2.50 | 2.36 |  |
|  | Total hits Pre | UDD | 14.84 | 4.55 | ***0.002*** | 14.23 | 4.99 | ***0.014*** | 15.24 | 4.26 | ***0.046*** | 14.24 | 4.63 | ***0.009*** | 15.81 | 4.33 | ***0.049*** |
|  |  | BDD | 15.53 | 4.94 |  | 15.47 | 4.98 |  | 15.60 | 4.99 |  | 15.77 | 4.92 |  | 15.00 | 5.09 |  |
|  | Total hits Post | UDD | 17.31 | 5.55 | ***0.018*** | 17.43 | 5.86 | 0.502 | 17.24 | 5.44 | ***0.001*** | 17.24 | 6.05 | 0.233 | 17.41 | 4.84 | ***0.008*** |
|  |  | BDD | 17.27 | 5.47 |  | 16.31 | 6.35 |  | 18.30 | 4.23 |  | 16.88 | 5.97 |  | 18.16 | 4.15 |  |
|  | Total misses Pre | UDD | 12.36 | 4.54 | ***0.001*** | 13.23 | 4.99 | ***0.016*** | 11.79 | 4.21 | ***0.027*** | 13.06 | 4.64 | ***0.010*** | 11.27 | 4.27 | ***0.022*** |
|  |  | BDD | 11.44 | 4.91 |  | 11.47 | 4.91 |  | 11.40 | 4.99 |  | 11.20 | 4.86 |  | 12.00 | 5.09 |  |
|  | Total misses Post | UDD | 9.84 | 5.61 | ***0.009*** | 10.00 | 6.06 | 0.331 | 9.74 | 5.40 | ***0.001*** | 10.03 | 6.17 | 0.138 | 9.55 | 4.76 | ***0.007*** |
|  |  | BDD | 9.56 | 5.43 |  | 10.35 | 6.28 |  | 8.67 | 4.19 |  | 9.89 | 5.91 |  | 8.79 | 4.09 |  |
|  | | | | | | | | | | | | | | | | | |
|  |  |  |  |  |  |  |  |  |  |  |  |  |  |  |  |  |  |
| **SWM** | SWM1_PRE | UDD | 33.86 | 21.60 | ***<0.001*** | 33.36 | 20.48 | ***0.004*** | 34.18 | 22.60 | ***0.001*** | 32.35 | 19.72 | ***<0.001*** | 36.18 | 24.54 | ***0.015*** |
|  |  | BDD | 30.11 | 21.78 |  | 35.94 | 22.62 |  | 23.50 | 19.06 |  | 32.69 | 22.17 |  | 24.00 | 20.09 |  |
|  | SWM1_POST | UDD | 22.93 | 17.48 | ***0.009*** | 22.68 | 16.97 | ***0.002*** | 23.09 | 18.05 | 0.846 | 20.74 | 16.33 | ***0.006*** | 26.32 | 19.00 | 0.707 |
|  |  | BDD | 24.47 | 16.83 |  | 24.94 | 16.15 |  | 23.93 | 17.84 |  | 25.18 | 16.54 |  | 22.79 | 17.84 |  |
|  | SWM2_PRE | UDD | 1.82 | 4.47 | ***0.025*** | 0.64 | 1.87 | >0.999 | 2.61 | 5.46 | ***0.020*** | 0.85 | 2.03 | 0.206 | 3.38 | 6.55 | 0.054 |
|  |  | BDD | 1.73 | 2.95 |  | 2.26 | 3.23 |  | 1.13 | 2.50 |  | 1.98 | 3.18 |  | 1.16 | 2.27 |  |
|  | SWM2_POST | UDD | 0.46 | 1.28 | 0.052 | 0.64 | 1.36 | 0.057 | 0.35 | 1.23 | 0.636 | 0.44 | 1.13 | 0.051 | 0.50 | 1.50 | 0.725 |
|  |  | BDD | 1.08 | 2.08 |  | 1.15 | 1.96 |  | 1.00 | 2.24 |  | 1.11 | 2.22 |  | 1.00 | 1.76 |  |
|  | SWM3_PRE | UDD | 9.12 | 8.16 | ***0.006*** | 11.00 | 9.30 | ***0.004*** | 8.11 | 7.47 | 0.295 | 10.52 | 8.49 | ***0.001*** | 7.17 | 7.47 | 0.872 |
|  |  | BDD | 9.35 | 8.27 |  | 10.79 | 8.90 |  | 7.67 | 7.30 |  | 9.69 | 8.71 |  | 8.65 | 7.48 |  |
|  | SWM3_POST | UDD | 6.07 | 5.98 | ***0.002*** | 4.61 | 6.19 | ***0.003*** | 7.00 | 5.75 | 0.227 | 5.15 | 5.78 | ***0.004*** | 7.37 | 6.18 | 0.228 |
|  |  | BDD | 6.32 | 6.00 |  | 6.11 | 5.73 |  | 6.57 | 6.43 |  | 6.11 | 5.77 |  | 6.92 | 6.84 |  |
|  | SWM4_PRE | UDD | 23.14 | 12.76 | ***0.015*** | 23.87 | 14.75 | ***0.038*** | 22.75 | 11.83 | 0.115 | 22.88 | 13.47 | ***0.030*** | 23.50 | 12.07 | 0.288 |
|  |  | BDD | 18.84 | 13.17 |  | 22.44 | 13.41 |  | 14.79 | 11.89 |  | 20.82 | 13.17 |  | 14.88 | 12.62 |  |
|  | SWM4_POST | UDD | 17.72 | 11.70 | 0.577 | 17.44 | 10.75 | 0.194 | 17.89 | 12.46 | 0.582 | 16.22 | 11.46 | 0.463 | 19.84 | 12.01 | 0.906 |
|  |  | BDD | 18.44 | 11.23 |  | 18.81 | 10.27 |  | 18.00 | 12.48 |  | 18.89 | 10.73 |  | 17.15 | 12.91 |  |
|  | SWM5_PRE | UDD | 3.70 | 4.31 | ***0.032*** | 3.73 | 3.71 | 0.060 | 3.69 | 4.74 | 0.160 | 3.00 | 3.36 | 0.297 | 4.90 | 5.47 | 0.055 |
|  |  | BDD | 4.44 | 8.70 |  | 5.41 | 9.90 |  | 3.33 | 7.10 |  | 5.53 | 10.08 |  | 1.84 | 2.50 |  |
|  | SWM5_POST | UDD | 2.54 | 3.64 | 0.204 | 2.36 | 2.61 | 0.260 | 2.65 | 4.21 | 0.575 | 2.29 | 3.53 | 0.111 | 2.91 | 3.87 | 0.289 |
|  |  | BDD | 3.11 | 4.12 |  | 3.44 | 4.53 |  | 2.73 | 3.64 |  | 3.24 | 4.42 |  | 2.79 | 3.41 |  |
|  | SWM6_PRE | UDD | 0.23 | 0.61 | 0.132 | 0.27 | 0.70 | 0.083 | 0.19 | 0.54 | 0.625 | 0.24 | 0.65 | 0.160 | 0.21 | 0.54 | 0.542 |
|  |  | BDD | 0.14 | 0.53 |  | 0.18 | 0.63 |  | 0.10 | 0.40 |  | 0.13 | 0.55 |  | 0.16 | 0.50 |  |
|  | SWM6_POST | UDD | 0.09 | 0.35 | 0.347 | 0.00 | 0.00 | 0.823 | 0.00 | 0.00 | 0.283 | 0.00 | 0.00 | 0.822 | 0.00 | 0.00 | 0.287 |
|  |  | BDD | 0.24 | 0.69 |  | 0.00 | 0.00 |  | 0.00 | 1.00 |  | 0.00 | 0.00 |  | 0.00 | 1.00 |  |
|  | SWM7_PRE | UDD | 0.74 | 1.66 | 0.886 | 1.27 | 2.15 | 0.210 | 0.46 | 1.29 | 0.229 | 1.04 | 2.03 | 0.582 | 0.33 | 0.84 | 0.132 |
|  |  | BDD | 1.56 | 3.71 |  | 1.75 | 4.70 |  | 1.33 | 2.12 |  | 1.80 | 4.42 |  | 1.06 | 1.43 |  |
|  | SWM7_POST | UDD | 0.72 | 1.33 | 0.284 | 0.00 | 1.00 | 0.612 | 1.00 | 1.00 | 0.205 | 1.00 | 2.00 | 0.288 | 1.00 | 1.00 | 0.884 |
|  |  | BDD | 0.80 | 1.50 |  | 1.00 | 2.00 |  | 1.00 | 1.00 |  | 1.00 | 1.00 |  | 1.00 | 2.00 |  |
|  | SWM8_PRE | UDD | 2.63 | 3.15 | 0.485 | 2.40 | 2.67 | 0.306 | 2.75 | 3.43 | 0.770 | 1.88 | 2.26 | 0.677 | 3.67 | 3.93 | 0.589 |
|  |  | BDD | 1.47 | 3.10 |  | 2.07 | 3.78 |  | 0.79 | 1.96 |  | 1.79 | 3.50 |  | 0.82 | 2.04 |  |
|  | SWM8_POST | UDD | 1.96 | 3.11 | 0.931 | 1.72 | 2.37 | 0.827 | 2.11 | 3.54 | 0.687 | 1.63 | 2.92 | 0.961 | 2.42 | 3.39 | 0.793 |
|  |  | BDD | 1.94 | 2.79 |  | 2.08 | 2.78 |  | 1.78 | 2.86 |  | 2.17 | 3.00 |  | 1.31 | 2.10 |  |
|  | SWM9_PRE | UDD | 2.28 | 3.35 | 0.107 | 2.00 | 2.33 | 0.372 | 2.47 | 3.93 | 0.185 | 1.62 | 2.12 | 0.821 | 3.40 | 4.63 | 0.056 |
|  |  | BDD | 1.97 | 3.29 |  | 2.30 | 3.94 |  | 1.60 | 2.39 |  | 2.32 | 3.72 |  | 1.16 | 1.77 |  |
|  | SWM9_POST | UDD | 1.55 | 2.75 | 0.810 | 1.45 | 2.34 | 0.566 | 1.62 | 3.02 | 0.669 | 1.50 | 2.99 | 0.323 | 1.64 | 2.40 | 0.214 |
|  |  | BDD | 1.86 | 2.46 |  | 1.88 | 2.53 |  | 1.83 | 2.42 |  | 1.76 | 2.34 |  | 2.11 | 2.79 |  |
|  | SWM10_PRE | UDD | 0.02 | 0.14 | 0.322 | 0.00 | 0.00 |  | 0.03 | 0.18 | 0.325 | 0.00 | 0.00 |  | 0.05 | 0.23 | 0.331 |
|  |  | BDD | 0.08 | 0.41 |  | 0.12 | 0.54 |  | 0.03 | 0.18 |  | 0.09 | 0.47 |  | 0.05 | 0.23 |  |
|  | SWM10_POST | UDD | 0.00 | 0.00 | 0.568 | 0.00 | 0.00 | 0.676 | 0.00 | 0.00 | 0.662 | 0.00 | 0.00 | 0.675 | 0.00 | 0.00 | 0.667 |
|  |  | BDD | 0.13 | 0.49 |  | 0.00 | 1.00 |  | 0.00 | 0.00 |  | 0.00 | 1.00 |  | 0.00 | 0.00 |  |
|  | SWM11_PRE | UDD | 0.49 | 1.20 | >0.999 | 0.80 | 1.61 | 0.179 | 0.32 | 0.90 | 0.242 | 0.68 | 1.46 | 0.600 | 0.22 | 0.65 | 0.207 |
|  |  | BDD | 0.64 | 1.61 |  | 0.35 | 1.23 |  | 0.96 | 1.92 |  | 0.58 | 1.87 |  | 0.76 | 0.97 |  |
|  | SWM11_POST | UDD | 0.50 | 1.03 | 0.601 | 0.00 | 1.00 | 0.731 | 1.00 | 1.00 | 0.336 | 1.00 | 1.00 | 0.581 | 0.00 | 1.00 | >0.999 |
|  |  | BDD | 0.54 | 1.15 |  | 0.00 | 1.00 |  | 1.00 | 1.00 |  | 0.00 | 1.00 |  | 1.00 | 1.00 |  |
|  | SWM12_PRE | UDD | 1.65 | 2.36 | 0.447 | 1.33 | 1.68 | 0.312 | 1.82 | 2.67 | 0.724 | 1.04 | 1.46 | 0.696 | 2.50 | 3.07 | 0.528 |
|  |  | BDD | 0.82 | 2.09 |  | 1.19 | 2.48 |  | 0.42 | 1.47 |  | 1.00 | 2.26 |  | 0.47 | 1.70 |  |
|  | SWM12_POST | UDD | 1.13 | 2.11 | 0.471 | 1.00 | 2.00 | 0.559 | 1.00 | 2.00 | 0.689 | 1.00 | 2.00 | 0.485 | 1.00 | 2.00 | 0.825 |
|  |  | BDD | 1.26 | 1.82 |  | 1.00 | 2.00 |  | 1.00 | 1.00 |  | 1.00 | 2.00 |  | 1.00 | 1.00 |  |
| **SWM** | SWM13_PRE | UDD | 35.25 | 23.18 | ***<0.001*** | 35.09 | 21.39 | ***0.004*** | 35.36 | 24.63 | ***0.004*** | 33.74 | 20.44 | ***<0.001*** | 37.71 | 27.41 | ***0.040*** |
|  |  | BDD | 32.06 | 22.80 |  | 38.09 | 24.11 |  | 25.23 | 19.41 |  | 35.18 | 23.32 |  | 24.68 | 20.18 |  |
|  | SWM13_POST | UDD | 23.95 | 18.12 | ***0.007*** | 23.68 | 17.31 | ***0.002*** | 24.12 | 18.89 | 0.900 | 21.59 | 16.85 | ***0.005*** | 27.59 | 19.77 | 0.745 |
|  |  | BDD | 25.78 | 17.47 |  | 26.53 | 17.07 |  | 24.93 | 18.16 |  | 26.69 | 17.20 |  | 23.63 | 18.37 |  |
|  | SWM14_PRE | UDD | 1.36 | 2.38 | ***0.005*** | 0.91 | 1.95 | 0.409 | 1.68 | 2.63 | ***0.007*** | 1.21 | 2.14 | ***0.033*** | 1.63 | 2.79 | 0.081 |
|  |  | BDD | 1.80 | 3.00 |  | 2.32 | 3.34 |  | 1.20 | 2.50 |  | 2.02 | 3.26 |  | 1.26 | 2.26 |  |
|  | SWM14_POST | UDD | 0.55 | 1.41 | 0.221 | 0.64 | 1.36 | 0.183 | 0.50 | 1.46 | >0.999 | 0.50 | 1.16 | 0.168 | 0.64 | 1.76 | 0.922 |
|  |  | BDD | 1.33 | 2.52 |  | 1.44 | 2.62 |  | 1.20 | 2.44 |  | 1.33 | 2.69 |  | 1.32 | 2.14 |  |
|  | SWM15_PRE | UDD | 9.37 | 8.37 | ***0.009*** | 11.47 | 9.65 | ***0.004*** | 8.25 | 7.54 | 0.377 | 10.88 | 8.77 | ***0.001*** | 7.28 | 7.51 | >0.999 |
|  |  | BDD | 9.81 | 8.86 |  | 11.32 | 9.84 |  | 8.04 | 7.36 |  | 10.23 | 9.46 |  | 8.94 | 7.68 |  |
|  | SWM15_POST | UDD | 6.28 | 6.19 | ***0.003*** | 4.78 | 6.49 | ***0.010*** | 7.25 | 5.91 | 0.167 | 5.33 | 6.11 | ***0.008*** | 7.63 | 6.22 | 0.231 |
|  |  | BDD | 6.58 | 6.17 |  | 6.48 | 5.89 |  | 6.70 | 6.62 |  | 6.41 | 5.89 |  | 7.08 | 7.12 |  |
|  | SWM16_PRE | UDD | 24.12 | 13.20 | ***0.018*** | 24.93 | 15.25 | ***0.045*** | 23.68 | 12.25 | 0.127 | 23.72 | 13.91 | ***0.035*** | 24.67 | 12.54 | 0.297 |
|  |  | BDD | 19.49 | 13.66 |  | 23.33 | 14.08 |  | 15.17 | 12.03 |  | 21.62 | 13.83 |  | 15.24 | 12.64 |  |
|  | SWM16_POST | UDD | 18.54 | 12.38 | 0.625 | 18.28 | 11.07 | 0.226 | 18.71 | 13.36 | 0.581 | 16.89 | 11.98 | 0.515 | 20.89 | 12.89 | 0.889 |
|  |  | BDD | 19.36 | 11.66 |  | 19.93 | 10.96 |  | 18.70 | 12.66 |  | 20.03 | 11.17 |  | 17.46 | 13.26 |  |
|  | SWM17_PRE | UDD | 35.02 | 4.56 | ***0.001*** | 35.36 | 3.81 | ***0.005*** | 34.79 | 5.03 | ***0.029*** | 35.18 | 3.97 | ***0.001*** | 34.77 | 5.43 | 0.285 |
|  |  | BDD | 33.59 | 6.35 |  | 33.85 | 7.32 |  | 33.30 | 5.15 |  | 33.80 | 6.55 |  | 33.11 | 5.99 |  |
|  | SWM17_POST | UDD | 32.91 | 4.80 | 0.166 | 33.23 | 3.78 | 0.641 | 32.71 | 5.41 | ***0.026*** | 32.44 | 4.22 | 0.519 | 33.64 | 5.62 | ***0.048*** |
|  |  | BDD | 32.58 | 4.93 |  | 33.26 | 4.57 |  | 31.80 | 5.29 |  | 33.18 | 4.48 |  | 31.16 | 5.75 |  |
|  | SWM18_PRE | UDD | 1364.41 | 521.66 | 0.062 | 1505.78 | 674.52 | ***0.014*** | 1272.93 | 376.92 | 0.857 | 1436.56 | 601.58 | 0.176 | 1252.90 | 350.10 | 0.115 |
|  |  | BDD | 1293.53 | 454.28 |  | 1262.15 | 429.34 |  | 1328.05 | 485.23 |  | 1306.99 | 419.75 |  | 1262.36 | 537.08 |  |
|  | SWM18_POST | UDD | 1249.60 | 469.46 | ***0.028*** | 1233.18 | 484.58 | 0.329 | 1260.23 | 466.47 | ***0.044*** | 1308.04 | 532.92 | ***0.043*** | 1159.28 | 341.84 | 0.334 |
|  |  | BDD | 1189.89 | 323.83 |  | 1211.95 | 312.68 |  | 1164.88 | 339.62 |  | 1202.11 | 286.32 |  | 1160.93 | 406.66 |  |
|  | SWM19_PRE | UDD | 1982.99 | 1230.17 | 0.222 | 2606.15 | 1736.44 | 0.108 | 1649.16 | 677.71 | 0.922 | 2210.68 | 1465.31 | 0.289 | 1666.76 | 724.84 | 0.564 |
|  |  | BDD | 1620.77 | 862.07 |  | 1634.30 | 952.16 |  | 1604.99 | 763.64 |  | 1624.06 | 856.91 |  | 1614.00 | 899.12 |  |
|  | SWM19_POST | UDD | 1692.42 | 939.90 | 0.854 | 1839.19 | 1036.38 | 0.695 | 1598.06 | 878.92 | 0.771 | 1818.70 | 1069.85 | 0.825 | 1512.96 | 705.24 | 0.979 |
|  |  | BDD | 1699.79 | 844.09 |  | 1594.77 | 759.53 |  | 1823.07 | 935.80 |  | 1700.10 | 794.50 |  | 1698.88 | 1007.79 |  |
|  | SWM20_PRE | UDD | 2414.69 | 1864.09 | 0.514 | 3364.30 | 2751.45 | 0.216 | 1905.97 | 842.92 | 0.436 | 2794.92 | 2291.91 | 0.324 | 1886.60 | 813.15 | 0.652 |
|  |  | BDD | 2306.31 | 1835.91 |  | 2440.90 | 2198.20 |  | 2149.28 | 1324.27 |  | 2353.03 | 1980.91 |  | 2210.12 | 1546.16 |  |
|  | SWM20_POST | UDD | 2108.65 | 1026.29 | 0.230 | 2319.31 | 1278.38 | 0.214 | 1973.23 | 823.33 | 0.991 | 2195.23 | 1189.27 | 0.247 | 1985.62 | 750.32 | 0.776 |
|  |  | BDD | 2073.77 | 1077.64 |  | 1940.55 | 878.30 |  | 2230.16 | 1275.74 |  | 1991.40 | 829.56 |  | 2308.21 | 1611.53 |  |
|  | SWM21_PRE | UDD | 15717.24 | 5112.15 | ***<0.001*** | 15639.43 | 4138.83 | ***0.011*** | 15769.11 | 5731.73 | ***0.003*** | 15451.08 | 3697.80 | ***0.003*** | 16148.17 | 6905.42 | ***0.009*** |
|  |  | BDD | 15589.12 | 5323.55 |  | 16068.88 | 4505.22 |  | 15045.38 | 6155.83 |  | 15668.67 | 4070.45 |  | 15400.71 | 7657.44 |  |
|  | SWM21_POST | UDD | 13128.77 | 3001.34 | ***<0.001*** | 13612.10 | 3672.93 | ***0.002*** | 12816.03 | 2484.37 | ***0.011*** | 13691.76 | 3484.94 | ***<0.001*** | 12258.70 | 1789.31 | 0.079 |
|  |  | BDD | 13333.50 | 2506.36 |  | 13700.80 | 2622.00 |  | 12917.22 | 2342.26 |  | 13425.50 | 2420.66 |  | 13115.59 | 2755.29 |  |
|  | SWM22_PRE | UDD | 26539.37 | 7377.62 | ***<0.001*** | 28855.09 | 8535.55 | ***<0.001*** | 25381.51 | 6588.05 | 0.150 | 28375.85 | 7728.96 | ***<0.001*** | 24090.72 | 6272.18 | 0.330 |
|  |  | BDD | 27275.26 | 11406.63 |  | 27999.12 | 12174.48 |  | 26430.77 | 10636.28 |  | 27136.44 | 11258.58 |  | 27561.07 | 12051.76 |  |
|  | SWM22_POST | UDD | 23520.97 | 5721.31 | ***0.024*** | 23546.11 | 6324.69 | 0.087 | 23504.81 | 5419.29 | 0.158 | 23941.20 | 6447.24 | 0.060 | 22923.80 | 4599.03 | 0.245 |
|  |  | BDD | 23818.28 | 5331.61 |  | 23539.14 | 4545.97 |  | 24145.96 | 6219.44 |  | 23560.19 | 4382.53 |  | 24552.83 | 7593.65 |  |
|  | SWM23_PRE | UDD | 43860.49 | 12491.83 | ***0.001*** | 48274.67 | 15663.72 | ***0.009*** | 41495.76 | 9944.57 | ***0.046*** | 46221.32 | 14280.00 | ***0.007*** | 40581.57 | 8835.18 | 0.097 |
|  |  | BDD | 40450.65 | 10934.87 |  | 42546.05 | 12367.57 |  | 38093.32 | 8722.22 |  | 41563.52 | 11495.72 |  | 38224.90 | 9654.14 |  |
|  | SWM23_POST | UDD | 38958.17 | 8510.01 | 0.536 | 40647.49 | 9430.71 | 0.480 | 37872.18 | 7847.08 | 0.980 | 39782.03 | 9581.98 | 0.555 | 37787.42 | 6779.75 | 0.859 |
|  |  | BDD | 40027.37 | 9599.46 |  | 40569.96 | 10021.75 |  | 39390.41 | 9260.70 |  | 40159.47 | 8999.60 |  | 39651.38 | 11536.57 |  |
|  | | | | | | | | | | | | | | | | | |
| **DMS** | A' Pre | UDD | 0.53 | 0.20 | 0.322 | 0.53 | 0.19 | 0.490 | 0.53 | 0.21 | 0.434 | 0.54 | 0.17 | 0.455 | 0.51 | 0.25 | 0.486 |
|  |  | BDD | 0.54 | 0.22 |  | 0.52 | 0.26 |  | 0.58 | 0.12 |  | 0.53 | 0.25 |  | 0.56 | 0.11 |  |
|  | A' Post | UDD | 0.48 | 0.21 | 0.806 | 0.48 | 0.26 | 0.651 | 0.48 | 0.18 | 0.057 | 0.45 | 0.24 | 0.651 | 0.55 | 0.13 | 0.057 |
|  |  | BDD | 0.60 | 0.17 |  | 0.57 | 0.20 |  | 0.63 | 0.13 |  | 0.58 | 0.18 |  | 0.64 | 0.13 |  |
|  | B" Pre | UDD | -0.54 | 0.54 | 0.124 | -0.50 | 0.55 | 0.359 | -0.57 | 0.53 | 0.222 | -0.55 | 0.53 | 0.446 | -0.52 | 0.56 | 0.118 |
|  |  | BDD | -0.67 | 0.52 |  | -0.56 | 0.57 |  | -0.80 | 0.43 |  | -0.63 | 0.55 |  | -0.76 | 0.46 |  |
|  | B" Post | UDD | -0.66 | 0.49 | 0.270 | -0.64 | 0.55 | 0.932 | -0.68 | 0.46 | 0.100 | -0.62 | 0.52 | 0.491 | -0.73 | 0.47 | 0.337 |
|  |  | BDD | -0.58 | 0.56 |  | -0.58 | 0.55 |  | -0.57 | 0.59 |  | -0.56 | 0.56 |  | -0.63 | 0.58 |  |
|  | Mean latency Pre | UDD | 3165.72 | 898.93 | 0.105 | 3261.66 | 809.57 | 0.757 | 3103.65 | 958.96 | 0.062 | 3166.80 | 869.39 | 0.386 | 3164.06 | 963.65 | 0.136 |
|  |  | BDD | 3336.79 | 936.75 |  | 3307.95 | 816.26 |  | 3368.50 | 1067.16 |  | 3375.31 | 889.86 |  | 3247.58 | 1057.73 |  |
|  | Mean latency Post | UDD | 3010.29 | 790.59 | 0.258 | 3210.97 | 714.62 | 0.348 | 2880.44 | 820.08 | 0.485 | 3057.60 | 794.27 | 0.070 | 2937.18 | 797.75 | 0.785 |
|  |  | BDD | 3237.05 | 832.57 |  | 3215.15 | 881.94 |  | 3261.87 | 787.12 |  | 3208.90 | 859.93 |  | 3303.73 | 782.20 |  |
|  | Mean latency (all delays) Pre | UDD | 3320.43 | 1020.23 | 0.129 | 3428.40 | 913.85 | 0.848 | 3250.57 | 1091.12 | 0.074 | 3325.00 | 984.89 | 0.413 | 3313.38 | 1096.22 | 0.176 |
|  |  | BDD | 3518.91 | 966.35 |  | 3415.27 | 884.70 |  | 3632.91 | 1052.21 |  | 3491.37 | 985.57 |  | 3582.67 | 943.35 |  |
|  | Mean latency (all delays) Post | UDD | 3148.93 | 852.74 | 0.249 | 3393.10 | 810.72 | 0.755 | 2990.94 | 853.35 | 0.212 | 3207.48 | 877.04 | 0.276 | 3058.44 | 825.60 | 0.648 |
|  |  | BDD | 3408.96 | 952.00 |  | 3381.70 | 1017.62 |  | 3439.84 | 888.05 |  | 3374.70 | 1000.84 |  | 3490.10 | 844.79 |  |
|  | Mean correct latency(simultaneous) Pre | UDD | 2761.71 | 650.21 | 0.204 | 2835.95 | 569.01 | 0.442 | 2713.67 | 701.81 | 0.320 | 2750.80 | 650.04 | 0.377 | 2778.56 | 665.41 | 0.366 |
|  |  | BDD | 3011.47 | 725.99 |  | 3014.87 | 754.18 |  | 3007.73 | 706.53 |  | 3048.44 | 765.00 |  | 2925.85 | 637.45 |  |
|  | Mean correct latency(simultaneous) Post | UDD | 2655.16 | 815.85 | ***0.006*** | 2722.39 | 660.57 | 0.079 | 2611.66 | 909.24 | ***0.034*** | 2653.42 | 791.44 | ***0.009*** | 2657.86 | 871.19 | 0.339 |
|  |  | BDD | 2802.70 | 708.71 |  | 2811.53 | 720.16 |  | 2792.70 | 707.65 |  | 2802.23 | 687.67 |  | 2803.83 | 775.88 |  |
|  | Percent correct Pre | UDD | 86.13 | 8.03 | 0.135 | 85.32 | 8.93 | 0.350 | 86.66 | 7.48 | 0.250 | 85.65 | 7.93 | 0.158 | 86.89 | 8.31 | 0.624 |
|  |  | BDD | 87.82 | 8.76 |  | 86.97 | 8.56 |  | 88.75 | 9.02 |  | 87.95 | 8.56 |  | 87.50 | 9.43 |  |
|  | Percent correct Post | UDD | 87.72 | 7.92 | 0.430 | 87.16 | 7.08 | 0.246 | 88.09 | 8.51 | 0.569 | 87.87 | 7.52 | 0.237 | 87.50 | 8.69 | 0.345 |
|  |  | BDD | 86.67 | 12.69 |  | 84.03 | 16.57 |  | 89.67 | 4.44 |  | 85.54 | 14.74 |  | 89.34 | 4.63 |  |
|  | Percent correct (all delays) Pre | UDD | 83.10 | 9.76 | 0.163 | 81.82 | 10.43 | 0.276 | 83.92 | 9.37 | 0.396 | 83.04 | 9.62 | 0.233 | 83.18 | 10.21 | 0.481 |
|  |  | BDD | 85.08 | 11.45 |  | 83.84 | 11.96 |  | 86.44 | 10.90 |  | 85.00 | 11.63 |  | 85.26 | 11.35 |  |
|  | Percent correct (all delays) Post | UDD | 84.94 | 10.05 | >0.999 | 84.55 | 8.76 | 0.576 | 85.20 | 10.92 | 0.574 | 85.39 | 9.32 | 0.685 | 84.24 | 11.28 | 0.477 |
|  |  | BDD | 85.66 | 10.38 |  | 83.43 | 12.78 |  | 88.28 | 5.75 |  | 84.74 | 11.62 |  | 87.96 | 5.96 |  |
|  | Percent false(simultaneous) Pre | UDD | 94.46 | 7.37 | 0.151 | 95.45 | 6.71 | 0.771 | 93.82 | 7.79 | 0.058 | 93.24 | 8.06 | 0.182 | 96.36 | 5.81 | 0.576 |
|  |  | BDD | 96.03 | 6.61 |  | 96.36 | 4.89 |  | 95.67 | 8.17 |  | 96.82 | 4.71 |  | 94.21 | 9.61 |  |
|  | Percent false(simultaneous) Post | UDD | 96.07 | 6.79 | 0.289 | 95.00 | 5.98 | 0.500 | 96.76 | 7.27 | 0.420 | 95.29 | 7.06 | 0.197 | 97.27 | 6.31 | 0.853 |
|  |  | BDD | 94.84 | 6.66 |  | 95.59 | 5.61 |  | 94.00 | 7.70 |  | 95.33 | 5.88 |  | 93.68 | 8.31 |  |
|  | Prob error given correct Pre | UDD | 0.14 | 0.08 | 0.310 | 0.15 | 0.09 | 0.696 | 0.14 | 0.07 | 0.291 | 0.15 | 0.08 | 0.355 | 0.14 | 0.08 | 0.692 |
|  |  | BDD | 0.13 | 0.09 |  | 0.13 | 0.09 |  | 0.12 | 0.10 |  | 0.13 | 0.09 |  | 0.14 | 0.10 |  |
|  | Prob error given correct Post | UDD | 0.13 | 0.09 | 0.744 | 0.14 | 0.09 | 0.532 | 0.13 | 0.09 | 0.309 | 0.13 | 0.09 | 0.619 | 0.13 | 0.09 | 0.196 |
|  |  | BDD | 0.12 | 0.08 |  | 0.14 | 0.11 |  | 0.10 | 0.05 |  | 0.13 | 0.10 |  | 0.11 | 0.05 |  |
|  | Prob error given error Pre | UDD | 0.11 | 0.13 | 0.130 | 0.13 | 0.15 | 0.268 | 0.09 | 0.12 | 0.318 | 0.11 | 0.14 | 0.302 | 0.11 | 0.13 | 0.244 |
|  |  | BDD | 0.09 | 0.15 |  | 0.11 | 0.15 |  | 0.07 | 0.13 |  | 0.10 | 0.15 |  | 0.08 | 0.14 |  |
|  | Prob error given error Post | UDD | 0.08 | 0.13 | 0.480 | 0.09 | 0.14 | 0.759 | 0.07 | 0.12 | 0.505 | 0.08 | 0.13 | 0.622 | 0.07 | 0.13 | 0.619 |
|  |  | BDD | 0.11 | 0.15 |  | 0.11 | 0.16 |  | 0.10 | 0.15 |  | 0.11 | 0.16 |  | 0.09 | 0.16 |  |
|  | Total correct Pre | UDD | 34.38 | 3.14 | 0.093 | 34.09 | 3.56 | 0.327 | 34.56 | 2.87 | 0.172 | 34.24 | 3.16 | 0.147 | 34.59 | 3.16 | 0.414 |
|  |  | BDD | 35.13 | 3.50 |  | 34.79 | 3.43 |  | 35.50 | 3.61 |  | 35.18 | 3.43 |  | 35.00 | 3.77 |  |
|  | Total correct Post | UDD | 35.09 | 3.17 | 0.968 | 34.86 | 2.83 | 0.455 | 35.24 | 3.40 | 0.569 | 35.15 | 3.01 | 0.458 | 35.00 | 3.48 | 0.345 |
|  |  | BDD | 35.19 | 3.23 |  | 34.59 | 4.05 |  | 35.87 | 1.78 |  | 34.96 | 3.66 |  | 35.74 | 1.85 |  |
|  | Total correct (all delays) Pre | UDD | 24.93 | 2.93 | 0.163 | 24.55 | 3.13 | 0.276 | 25.18 | 2.81 | 0.396 | 24.91 | 2.89 | 0.233 | 24.95 | 3.06 | 0.481 |
|  |  | BDD | 25.52 | 3.44 |  | 25.15 | 3.59 |  | 25.93 | 3.27 |  | 25.50 | 3.49 |  | 25.58 | 3.40 |  |
|  | Total correct (all delays) Post | UDD | 25.48 | 3.02 | 0.767 | 25.36 | 2.63 | 0.576 | 25.56 | 3.28 | 0.360 | 25.62 | 2.80 | 0.685 | 25.27 | 3.38 | 0.256 |
|  |  | BDD | 25.70 | 3.09 |  | 25.03 | 3.83 |  | 26.47 | 1.70 |  | 25.42 | 3.49 |  | 26.37 | 1.74 |  |
|  | Total correct(simultaneous) Pre | UDD | 9.45 | 0.74 | 0.151 | 9.55 | 0.67 | 0.771 | 9.38 | 0.78 | 0.058 | 9.32 | 0.81 | 0.182 | 9.64 | 0.58 | 0.576 |
|  |  | BDD | 9.60 | 0.66 |  | 9.64 | 0.49 |  | 9.57 | 0.82 |  | 9.68 | 0.47 |  | 9.42 | 0.96 |  |
|  | Total correct(simultaneous) Post | UDD | 9.61 | 0.68 | 0.289 | 9.50 | 0.60 | 0.500 | 9.68 | 0.73 | 0.420 | 9.53 | 0.71 | 0.197 | 9.73 | 0.63 | 0.853 |
|  |  | BDD | 9.48 | 0.67 |  | 9.56 | 0.56 |  | 9.40 | 0.77 |  | 9.53 | 0.59 |  | 9.37 | 0.83 |  |
| **OTS** | Mean choices to correct Pre | UDD | 1.23 | 0.26 | 0.281 | 1.21 | 0.16 | 0.658 | 1.25 | 0.31 | 0.188 | 1.26 | 0.19 | 0.096 | 1.19 | 0.35 | 0.848 |
|  |  | BDD | 1.26 | 0.21 |  | 1.26 | 0.22 |  | 1.26 | 0.21 |  | 1.27 | 0.23 |  | 1.23 | 0.18 |  |
|  | Mean choices to correct Post | UDD | 1.21 | 0.19 | ***<0.001*** | 1.24 | 0.21 | ***0.002*** | 1.19 | 0.17 | ***0.022*** | 1.23 | 0.19 | ***0.001*** | 1.19 | 0.17 | 0.068 |
|  |  | BDD | 1.17 | 0.17 |  | 1.15 | 0.17 |  | 1.19 | 0.17 |  | 1.17 | 0.18 |  | 1.16 | 0.16 |  |
|  | Mean latency to correct Pre | UDD | 11164.57 | 4670.93 | ***<0.001*** | 11059.11 | 4473.45 | ***0.003*** | 11231.68 | 4859.63 | ***0.003*** | 11078.26 | 4088.04 | ***0.002*** | 11300.21 | 5571.86 | ***0.009*** |
|  |  | BDD | 11952.88 | 4091.65 |  | 12037.60 | 4163.59 |  | 11856.86 | 4077.38 |  | 12076.51 | 3846.24 |  | 11660.07 | 4722.79 |  |
|  | Mean latency to correct Post | UDD | 9250.57 | 3741.07 | ***<0.001*** | 9364.15 | 3719.45 | ***<0.001*** | 9174.85 | 3811.04 | ***0.001*** | 9505.16 | 3744.67 | ***<0.001*** | 8838.38 | 3789.76 | ***0.007*** |
|  |  | BDD | 9147.70 | 3084.53 |  | 9375.68 | 2433.79 |  | 8889.32 | 3714.66 |  | 9575.61 | 2579.91 |  | 8134.21 | 3935.50 |  |
|  | Mean latency to first choice Pre | UDD | 10095.96 | 3931.12 | ***<0.001*** | 10313.24 | 4074.08 | ***0.004*** | 9951.11 | 3889.92 | ***0.004*** | 10241.18 | 3773.78 | ***0.002*** | 9860.85 | 4258.26 | ***0.009*** |
|  |  | BDD | 10489.94 | 3616.56 |  | 10623.52 | 3560.68 |  | 10347.46 | 3730.74 |  | 10640.58 | 3326.14 |  | 10149.03 | 4281.80 |  |
|  | Mean latency to first choice Post | UDD | 8292.87 | 3066.07 | ***<0.001*** | 8340.67 | 3129.24 | ***0.002*** | 8261.00 | 3071.66 | ***0.008*** | 8579.71 | 3335.38 | ***<0.001*** | 7828.46 | 2580.66 | ***0.042*** |
|  |  | BDD | 8602.05 | 2514.46 |  | 8684.65 | 2278.11 |  | 8513.93 | 2781.33 |  | 8737.43 | 2393.44 |  | 8295.66 | 2813.68 |  |
|  | Problems solved on first choice Pre | UDD | 16.30 | 3.01 | 0.054 | 16.55 | 3.13 | 0.928 | 16.15 | 2.97 | ***0.019*** | 16.18 | 2.89 | 0.102 | 16.50 | 3.25 | 0.304 |
|  |  | BDD | 16.36 | 2.59 |  | 16.24 | 2.75 |  | 16.50 | 2.43 |  | 16.20 | 2.73 |  | 16.74 | 2.26 |  |
|  | Problems solved on first choice Post | UDD | 16.93 | 2.26 | ***0.002*** | 16.59 | 2.32 | ***<0.001*** | 17.15 | 2.23 | 0.491 | 16.82 | 2.14 | ***<0.001*** | 17.09 | 2.49 | 0.705 |
|  |  | BDD | 17.33 | 2.17 |  | 17.79 | 1.74 |  | 16.79 | 2.51 |  | 17.49 | 1.95 |  | 16.94 | 2.67 |  |
